# SCD1/FADS2 fatty acid desaturases equipoise lipid metabolic activity and redox-driven ferroptosis in ascites-derived ovarian cancer cells

**DOI:** 10.7150/thno.70194

**Published:** 2022-04-24

**Authors:** Yang Xuan, Huogang Wang, Mingo MH Yung, Fushun Chen, Wai-Sun Chan, Yau-Sang Chan, Stephen KW Tsui, Hextan YS Ngan, Karen KL Chan, David W Chan

**Affiliations:** 1 Department of Obstetrics & Gynaecology, LKS Faculty of Medicine, The University of Hong Kong, Hong Kong SAR, P.R. China.; 2 School of Biomedical Sciences, The Chinese University of Hong Kong, Hong Kong SAR, People's Republic of China.

**Keywords:** ovarian cancer, peritoneal metastases, lipid metabolism, lipid desaturases, oxidative stress

## Abstract

**Rationale:** Malignant ascites in peritoneal metastases is a lipid-enriched microenvironment and is frequently involved in the poor prognosis of epithelial ovarian cancer (EOC). However, the detailed mechanisms underlying ovarian cancer (OvCa) cells dictating their lipid metabolic activities in promoting tumor progression remain elusive.

**Methods:** The omental conditioned medium (OCM) was established to imitate the omental or ascites microenvironment. Mass spectrometry, RT-qPCR, IHC, and western blot assays were applied to evaluate human fatty acid desaturases expressions and activities. Pharmaceutical inhibition and genetic ablation of SCD1/FADS2 were performed to observe the oncogenic capacities. RNA sequencing, lipid peroxidation, cellular iron, ROS, and Mito-Stress assays were applied to examine ferroptosis. OvCa patient-derived organoid and mouse model of peritoneal metastases were used to evaluate the combined effect of SCD1/FADS2 inhibitors with cisplatin.

**Results:** We found that two critical fatty acid desaturases, stearoyl-CoA desaturase-1 (SCD1) and acyl-CoA 6-desaturase (FADS2), were aberrantly upregulated, accelerating lipid metabolic activities and tumor aggressiveness of ascites-derived OvCa cells. Lipidomic analysis revealed that the elevation of unsaturated fatty acids (UFAs) was positively associated with SCD1/FADS2 levels and the oncogenic capacities of OvCa cells. In contrast, pharmaceutical inhibition and genetic ablation of SCD1/FADS2 retarded tumor growth, cancer stem cell (CSC) formation and reduced platinum resistance. Inhibition of SCD1/FADS2 directly downregulated GPX4 and the GSH/GSSG ratio, causing disruption of the cellular/mitochondrial redox balance and subsequently, iron-mediated lipid peroxidation and mitochondrial dysfunction in ascites-derived OvCa cells.

**Conclusions:** Combinational treatment with SCD1/FADS2 inhibitors and cisplatin synergistically repressed tumor cell dissemination, providing a promising chemotherapeutic strategy against EOC peritoneal metastases.

## Introduction

Metastasis is the common cause of high mortality in most cancers and accounts for 90% of all cancer deaths 1. In epithelial ovarian cancer (EOC), peritoneal metastases or peritoneal carcinomatosis are the most common form of cancer cell spreads and is significantly correlated with a poor prognosis 2, 3. Emerging evidence has indicated that malignant ascites act as a distinct tumor microenvironment providing plenty of cytokines, growth factors, and bioactive lipids for governing tumor development and metastatic progression 4-6. Notably, the high lipid content plays an essential role in modulating ovarian cancer (OvCa) cell survival, proliferation, and invasion during tumor colonization, supporting the rationale for preferential dissemination of OvCa cells to distal sites with a lipid-enriched microenvironment 7, 8. We and others have recently shown that both the survival and metastatic capacities of cancer cells are determined by *de novo* fatty acid synthesis and fatty acid β-oxidation (FAO) via uptake of free fatty acids from the malignant ascites or omental microenvironment during EOC peritoneal metastases 6, 7, 9. Notably, the activities of fatty acid desaturation are also concomitantly elevated and are crucially involved in maintaining the stemness and aggressiveness of numerous malignancies 10, 11. Indeed, the aberrant upregulation of SCD1 and/or FADS2, the key fatty acid desaturases, are commonly found in cancers with high aggressiveness and chemoresistance 9, 10. Regardless of these critical observations, there has been a lack of in-depth investigations in deciphering the molecular mechanisms underlying these critical fatty acid desaturases in governing the metastatic progression of ascites-derived OvCa cells in peritoneal metastases.

Here, we report that OvCa cells derived from the malignant ascites, rather than primary tumors had relatively enhanced fatty acid desaturation activities accompanied by the upregulation of SCD1 and FADS2 fatty acid desaturases. Functional studies revealed that SCD1/FADS2 were positively associated with cell proliferation, cell migration, and tumor growth of OvCa cells while suppressed G1/S cell cycle arrest, oxidative stress, and cell death in the lipid-enriched microenvironment. Notably, the study herein novelistically identified that SCD1/FADS2 were associated with redox equilibrium and lipid peroxidation, synergistic combinations of cisplatin and ferroptosis inducers enhanced chemotherapeutic efficiency and eradicated peritoneal metastasis of EOC.

## Methods

### Cell culture and human clinical samples

PEO1, PEO4, THP-1, OVCAR-3, OVSAHO, and OVKATE cells were cultured in RPMI-1640 medium, OVCA433, COV318, A2780s, and A2780cp cells were cultured in Dulbecco's Modified Eagle's medium, ES-2 and SK-OV-3 cells were cultured in McCoy's 5a Modified medium, and T-HESCs were cultured in a 1:1 mixture of Dulbecco's modified Eagle's medium and Ham's F-12 medium. HOSE cell lines were cultured in Ovarian Epithelial Cell Medium. All the media were supplemented with 10% FBS and 100 IU/mL penicillin. All the cells were cultured in an incubator (5% CO_2_, 37 °C). All the cell lines were subjected to cell line authentication tests (except HOSEs and ID8, a murine high-grade OvCa cell line, because of lacking their STR reference profiles) and mycoplasma-free testing. All the clinical samples (tumor, omentum, and ascites from OvCa patients) were obtained from the Queen Mary Hospital with prior approval of the Institutional Review Board of the University of Hong Kong/Hospital Authority Hong Kong West Cluster (HKU/HA HKW IRS) (IRS Reference Number: UW 11-298 and UW 20-256).

### Tumor tissue digestion, patient-derived organoid culture, and EMT model

For tumor digestion, according to the protocol 12, fresh tumor tissues were cut into small pieces (2 mm), digested with 2.5 mg/mL collagenase type II and DNAse, and shaken at 37 °C for 30 mins. Samples were then filtered (10 µm, Falcon) to a single-cell suspension. For OvCa organoid culture, cells were mixed with growth factor-reduced Matrigel (Corning). The mixed suspension was then rapidly plated into a 24-well plate. General culture medium contained Advanced DMEM/F12, penicillin-streptomycin (1%), GlutaMAX™ Supplement (1x), HEPES (1%), R-Spondin 1(100 ng/mL), Noggin (100 ng/mL), EGF (50 ng/mL), FGF-10 (10 ng/mL), FGF-2 (10 ng/mL), B27 (1x), nicotinamide (10 mM), N-acetycysteine (1.25 mM), prostaglandin E2 (1 µM), SB202190 (10 mM), A83-01 (500 nM), and Y-27632 dihydrochloride (10 µM). Organoid models were routinely tested for Mycoplasma and used to make stock in liquid nitrogen. For the organoid EMT model, the organoids were cultured in Matrigel and treated with TGF-β (5 ng/mL) to induce a spindle-like morphology, representing the EMT process.

### Plasmids and CRISPR/Cas9-mediated transfection

According to the Nature Protocols 13, both human and mouse SCD1 and FADS2 genes were designed as three independent sgRNAs for each gene from online tools (http://crispr.mit.edu/). The materials used in the construction contained the vector PX459 (pSpCas9(BB)-2A-Puro V2.0) and PX458(SpCas9-2A-GFP) plasmids, the transfected cells were selected by puromycin and identified through immunoblotting.

### Western blot analysis

Fresh cells were lysed with RIPA lysis buffer. Protein was separated by SDS-PAGE and transferred to FL-PVDF membranes. The secondary antibodies were prepared using LI-COR IRDye 6800/800 CW (1:20,000). Immunoblot imaging was performed using the LI-COR Odyssey CLx Imager.

### RNA extraction and RT-qPCR analysis

Total RNA was isolated by TRIzol reagent (Invitrogen). RNA was synthesized into cDNA using the SuperScript™ VILO™ cDNA Synthesis Kit (Thermo Fisher). TaqMan™ Universal PCR Master Mix was used to conduct the qPCR analysis. Real-Time PCR (ViiA7) was used to analyze the samples. The indicated mRNA was used for the normalization of gene expression in each experiment.

### Next-generation sequencing

The RNA samples were prepared well and sent to Genomic Center. The RNA quality was detected by an Agilent 4200 TapeStation.

### Mass spectrometry

OVCA433 and ES-2 cell lines were cultured in DMEM or fresh OCM medium. Ultra-performance liquid chromatography coupled to tandem-mass spectrometry (UPLC-MS/MS) system was performed in Metabo-Profile. LC-MS/MS analyses were processed by the company and analyzed in triplicates for each condition.

### Lipid quantification assay (Colorimetric)

OvCa cells with the indicated treatments were collected and washed with PBS. Lipids were extracted from OvCa cells using a Lipid Extraction Kit (Cell Biolabs). The UFAs in cancer cells were detected at OD 540 nm, and the values were calculated according to the Lipid Quantification Kit protocol.

### Determination of cell membrane fluidity

OvCa cells were seeded in 96-well microplates and treated with the indicated treatments for 48 h. Fluorescent Lipid Reagent (Abcam) and Pluronic F-127 were prepared to label the cell membrane. According to the membrane fluidity kit protocol, after incubation, washing of the cells and adding of media, the value was read by a fluorescence microplate reader or imaged by fluorescence microscopy 14.

### Confocal immunofluorescence assay

IF assays were performed in cells, organoids, and tumor tissues from humans. Cell lines and organoids were fixed with 4% paraformaldehyde for 15 min and then blocked with 5% BSA for 30 min. The primary antibodies were diluted in 1% BSA and incubated at 4 °C overnight. Then, secondary antibodies were added to the samples and incubated at room temperature for 1 hour. The cell nuclei were stained with DAPI. For tumor tissues, samples were soaked in xylene for 15 min twice and then washed with ethanol and anhydrously denatured according to a concentration gradient. Antigen retrieval was conducted with citrate (pH = 6.0), and samples were blocked. The primary antibodies were incubated with the samples at 4 °C overnight. The Opal™ Polaris 7 Color Automation IHC Detection Kit (Akoya) was used according to the protocol.

### Apoptosis assay

OvCa cells were treated with the indicated treatments for 48 h. For flow cytometry analysis, after the different treatments, cells were stained with an Alexa Fluor 488 Annexin V/Dead Cell Apoptosis Kit (Thermo Fisher) according to the protocol. For caspase-3/7 green detection, cells were stained with 5 µM CellEvent™ Caspase-3/7 Green Detection Reagent after indicated treatments. The cells were fixed and imaged by fluorescence microscopy.

### ROS assay

Cell antioxidant or ROS activity was detected by an OxiSelect™ Intracellular ROS Assay Kit (Cell Biolabs) according to the protocol. After the different treatments, the cells were cultured in 96-well plates and incubated in DCF dye loading, and then the fluorescence was quantified at 480 nm/530 nm. Mitochondrial ROS was detected with a MitoSOX^TM^ Staining Kit (Invitrogen) and Mito Tracker Green Staining Kit (Invitrogen). Cells from different groups were washed with PBS before incubation with a mixture of staining medium in the dark for 30 min, and then analyzed by fluorescence microscopy.

### Iron assay

According to the Iron Assay Kit (ScienCell) protocol, the procedure was detected in a 96-well plate. The iron standard was prepared, and the cell lysate was homogenized and diluted to ensure that the readings were within the standard curve range. Finally, the absorbance of the cells was read at 590 nm.

### C11-BODIPY for lipid peroxidation assay and GSH/GSSG determination

To evaluate lipid peroxidation or cellular GSH/GSSG in OvCa cells, the cells were treated with indicated treatments for 48 h. For confocal imaging, cell lipid peroxidation was detected by staining the fluorescent dye C11-BODIPY^581/591^ (Thermo Fisher) with the probe (2.5 µM) for 30 mins at 37 °C. Mounting media was used as an anti-fade reagent. Images were acquired using inverted microscopy (Carl Zeiss LSM 880). For flow cytometry, the cells were washed subsequent to staining with C11-BODIPY and analyzed by a BD LSRII flow cytometer (Becton Dickinson). The cellular GSH/GSSG ratios in OvCa cells were detected using the GSH/GSSG Ratio Detection Assay Kit II (Abcam, Fluorometric-Green) according to the manufacturer's instructions.

### Cell proliferation and Matrigel cell migration assays

Cell proliferation was evaluated using an XTT cell proliferation kit (Roche). Approximately 3000 cells were cultured in each well of a 96-well plate and incubated with indicated treatments. Finally, the absorbance was read at 492 nm. The migrated cells were stained by crystal violet and cell counted using light microscopy.

### *Ex vivo* studies

According to a previous OvCa *ex vivo* study^6^, ^15^, fresh immunocompetent C57/BL6 mouse omental fat was collected immediately after death and attached to the ultralow attached surface of Costar® 6-well clear TC-treated multiple well plates. GFP-labeled mouse ID8 cell lines were prepared, and 2 × 10^6^ cells/mL in DMEM/F12 (1:1) with 20% FBS was loaded with 500 µL of the single-cell suspension. After coculturing with the cell suspension for 24 h at 37 °C in a 5% CO_2_ incubator, the omentum was transferred to a new ultralow attachment plate, washed with PBS and replenished with new medium. The new medium was changed every three days. Images were acquired and analyzed using a ZOE^TM^ Fluorescent Imager.

### Animal study

According to our previous *in vivo* intraperitoneal mouse study 6, GFP-labeled ES-2 (5 × 10^5^ cells in 100 μl PBS) cells were intraperitoneally (i.p.) injected into 4- to 6-week-old female SCID mice. Different treatment was performed after tumor metastasis formation. The IVIS® Spectrum *In vivo* Imaging System (PerkinElmer) was used to evaluate cancer cell metastasis through injection of D-Luciferin (Gold Biotechnology) in the mouse intraperitoneal cavity. Metastatic tissues were examined by IHC staining. The entire mouse experimental design, animal maintenance and operational procedures were conducted in accordance with the animal license protocol approval of the Live Animals in Teaching and Research at the University of Hong Kong (CULATR number: 5466-20).

### Statistics and reproducibility

All the data were analyzed and graphed using GraphPad Prism software, or R studio. The experimental data are presented as the mean ± SEM of three independent trials. Two-group comparisons were analyzed by Student's two-sided t-test, and multiple group comparisons were analyzed by one-way ANOVA + Dunnett's two-sided test (when each group was compared with a control group) or one-way ANOVA + Tukey's two-sided test (when each group was compared with every other group).

## Results

### SCD1/FADS2 fatty acid desaturases are aberrantly upregulated in metastatic OvCa cells

**Figure 1:**
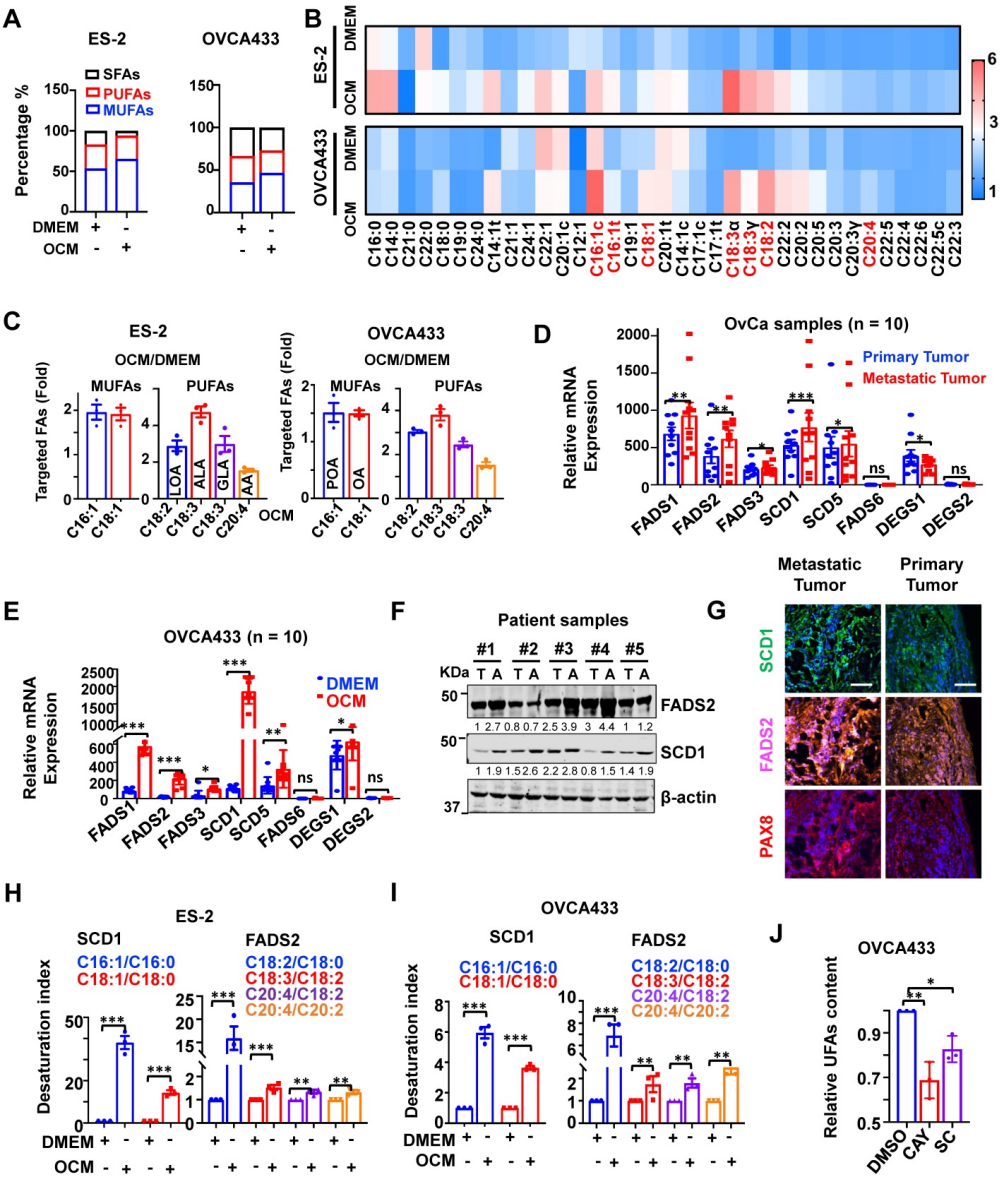
** SCD1 and FADS2 are activated and overexpressed in metastatic OvCa cells. (A-C)** Percentage bar chart **(A)**, heatmap **(B)**, and column scatter graph **(C)** show LC-MS/MS lipidomic analysis of the amount of intercellular SFAs, PUFAs, and MUFAs in OVCA433 and ES-2 cells, respectively. Cells were co-cultured in OCM for 12 h. 1% FBS-DMEM was used as the negative control. The experiments were conducted in three replicated sets of OCM, and the results are presented as the means of replicates. **(D)** mRNA levels of the indicated FA desaturases in paired primary and metastatic OvCa tissues (*n* = 10). 18S was used as an internal control. **(E)** mRNA levels of the indicated FA desaturases in OCM-co-cultured OVCA433 cells. OCM was established from 10 patients. 18S was used as an internal control. **(F)** Representative western blot and quantification analysis of indicated proteins in ascites tumor spheroids (A) and paired original tumors (T). β-actin was used as the internal control. **(G)** Representative fluorescent mIHC images of indicated proteins in 10 paired primary and omental metastatic OvCa tissue samples. Scale bar, 100 µm. **(H and I)** LC-MS/MS lipidomic analysis shows SCD1 and FADS2 desaturation indexes in OVCA433 and ES-2 cells. **(J)** UFA quantification after treatment with SCD1 inhibitor (CAY10566, 10 nM) or FADS2 inhibitor (sc26196, 100 nM) in OVCA433 cells for 48 h. Relative UFA quantification was normalized to the DMSO group. The results are representative findings of three independent experiments. Error bars represent the mean ± SEM. *P < 0.05, **P < 0.01, and ***P < 0.001.

This study utilized omental conditioned medium (OCM) to mimic the omental or ascites microenvironment and demonstrate that the cellular composition of UFAs, especially mono-UFAs (MUFAs), was significantly increased by approximately 12% in OvCa cell lines, such as ES-2 cells. This increase in MUFAs was intimately correlated with the loss of saturated-FAs (SFAs) in ES-2 cells co-cultured in OCM (Figure 1A-B). Under similar conditions, the composition of UFAs increased by approximately 6.5% in another OvCa cell line, OVCA433 cells, compared with the DMEM control (Figure 1A-B). Lipidomic analysis revealed that these UFAs primarily consisted of MUFAs, including palmitoleic acid (C16:1, POA) and oleic acid (C18:1, OA), and PUFAs, such as linoleic acid (C18:2, LOA), alpha-linolenic (C18:3, ALA), gamma-linolenic (C18:3, GLA) and arachidonic acid (C20:4, AA), in both OVCA433 and ES-2 cells (Figure 1C). QPCR analysis generally showed that relatively higher mRNA levels of *SCD1* and *FADS2* were detected in samples of omental metastatic tumors than in the respective primary tumors (*n* = 10 pairs) (Figure 1D). *SCD1* and *FADS2* levels were elevated by 16.13-fold and 11.34-fold, respectively, in OVCA433 cells with OCM (Figure 1E). Consistently, western blot analysis showed that both SCD1 and FADS2 were commonly upregulated in OvCa cell lines compared with human immortalized epithelial ovarian cells (HOSEs) (Figure S1A). Moreover, higher expression levels of SCD1 and FADS2 were also observed in spheroids derived from the malignant ascites of OvCa (Figure 1F). Of note, multiparametric immunohistochemical (IHC) analysis revealed that SCD1/FADS2 were remarkably upregulated in omental metastatic OvCa compared with their paired primary tumors (*n* = 10 pairs) (Figure 1G). Intriguingly, *in silico* analysis of the Cancer Genome Atlas (TCGA) showed that SCD1 and FADS2 were the major isoforms and were highly expressed in tumor tissues (Figure S1B), and were associated with poor overall survival (OS) (Figure S1C), advanced stage (Stage IV), and tumor recurrence (Figure S1D) in OvCa. Collectively, these findings show that SCD1 and FADS2 are aberrantly overexpressed in metastatic OvCa cells, especially in the lipid-enriched microenvironment.

### Upregulated SCD1 and FADS2 positively enhance fatty acid desaturase activities

Liquid chromatography with tandem mass spectrometry (LC-MS/MS) analysis demonstrated that both the SCD1 and FADS2 desaturation index were increased over ~14.33-fold and ~1.33-fold, respectively, in ES-2 cells co-cultured in OCM (Figure 1H). Similarly, in OVCA433 cells co-cultured with OCM, both the SCD1 and FADS2 desaturation index were increased more than ~3.65-fold and ~1.74-fold, respectively (Figure 1I), indicating concomitant upregulation in the expression and desaturase activities of both SCD1 and FADS2 in OvCa cells. To validate the vital functional role of both SCD1 and FADS2, selective inhibitors of SCD1, *e.g.,* CAY10566 and FADS2, *e.g.,* sc26196, were exploited, and the results of UFAs quantification assay (Colorimetric) showed that pharmaceutical inhibition of SCD1 or FADS2 led to 32% and 16% reductions in UFA content, respectively, in OVCA433 cells upon treatment with OCM (Figure 1J). These outcomes substantiate that the aberrant upregulation of SCD1 and FADS2 is the prominent FADS exhibiting fatty acid desaturase activities in OvCa cells derived from the lipid-enriched microenvironment.

### SCD1 and FADS2 are required for the oncogenic capacities of OvCa cells

**Figure 2:**
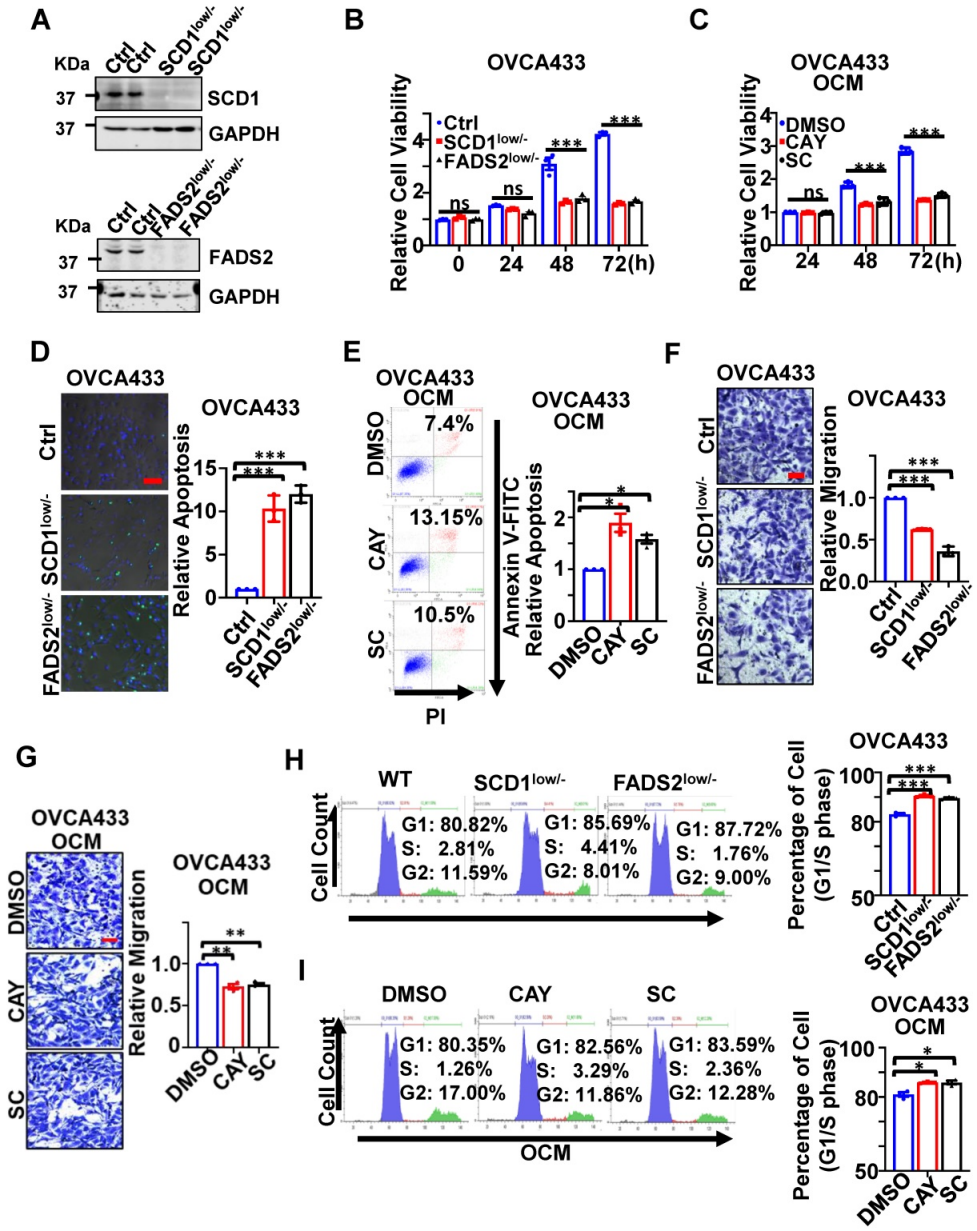
** SCD1/FADS2 deficiency inhibits OvCa propagation and invasion and promotes cell death. (A)** Representative western blot analysis of SCD1 and FADS2 in SCD1^low/-^ or FADS2^low/-^ clones of OVCA433 and ES-2 cells, same cells were found in Figure 3D. Ctrl = scrambled controls. Cells in** B, D, F, and H are** SCD1^low/-^ or FADS2^low/-^ clones of OVCA433 cells. OCM-co-cultured OVCA433 cells in **C, E, G, and I** were treated with SCD1 inhibitor (CAY10566, 10 nM) or FADS2 inhibitor (sc26196, 100 nM) for 48 h. **(B and C)** XTT cell proliferation analysis. **(D)** Representative caspase-3/7 fluorescent images. Nuclei were stained with Hoechst (blue). **(E)** Cell apoptosis was analyzed by flow cytometry. Cells were stained by Annexin V and propidium iodide (PI). **(F and G)** Representative images of the transwell migration assay. **(H and I)** Representative images of the cell cycle are determined by PI staining. Quantification in **(B-I)** is the mean ± SEM (*n* = 3 independent experiments). Statistical significance was determined by the two-tailed t-test. *P < 0.05, **P < 0.01, ***P < 0.001.

CRISPR/Cas9-mediated SCD1/FADS2 depletion was firstly established in OvCa cell lines, such as OVCA433 and ES-2 cells, to generate SCD1^low/-^ or FADS2^low/-^ clones to examine their functional roles (Figure 2A). Functionally, cell proliferation assay showed that silencing of SCD1 or FADS2 significantly hindered the cell growth of OVCA433 cells (Figure 2B) and ES-2 cells (Figure S2A) by ~2-fold upon three days of culture. The findings herein were in accordance with the effect of using SCD1/FADS2 inhibitors, in which OCM-mediated cell growth was remarkably repressed by at least 1.86-fold in OVCA433 cells compared with the controls (Figure 2C). In addition, sphere-formation capability was substantially inhibited by more than 1.6-fold in both SCD1^low/-^ and FADS2^low/-^ clones of OVCA433 and ES-2 cells when compared with respective controls (Ctrl) (Figure S2B).

On the other hand, apoptosis was markedly promoted in SCD1^low/-^ or FADS2^low/-^ clones of OVCA433 cells by 10.2-fold and 12.1-fold, respectively, compared with the controls (Figure 2D). Similar outcomes were observed in ES-2 SCD1^low/-^ cells and ES-2 FADS2^low/-^ cells, yielding a significant increase in cell apoptosis by 12.79-fold and 14.7-fold (Figure S2C) compared with control cells. Furthermore, apoptotic cell death was enhanced by 1.7-fold and 1.42-fold in OVCA433 cells upon treatment with SCD1/FADS2 selective inhibitors compared with the OCM cultured controls (Figure 2E). Conversely, migration assays showed an approximately 2-fold reduction in the migration of OVCA433 (Figure 2F) and ES-2 cells (Figure S2D) with SCD1/FADS2 knockout compared to the respective controls. Similarly, a 25% decrease in OCM-mediated cell migration was seen in OVCA433 cells treated with SCD1/FADS2 selective inhibitors compared with the controls (Figure 2G). Silencing or pharmacological inhibition of SCD1/FADS2 in OVCA433 (Figure 2H-I) and ES-2 cells (Figure S2E) correspondingly led to a small but significant increase in the G1/S cell cycle phase compared with the respective controls. Collectively, upregulation of SCD1/FADS2 expression and FA desaturation activities are accompanied by the pathogenesis of OvCa.

### Inhibition of SCD1/FADS2 impairs tumor initiation and membrane fluidity

**Figure 3:**
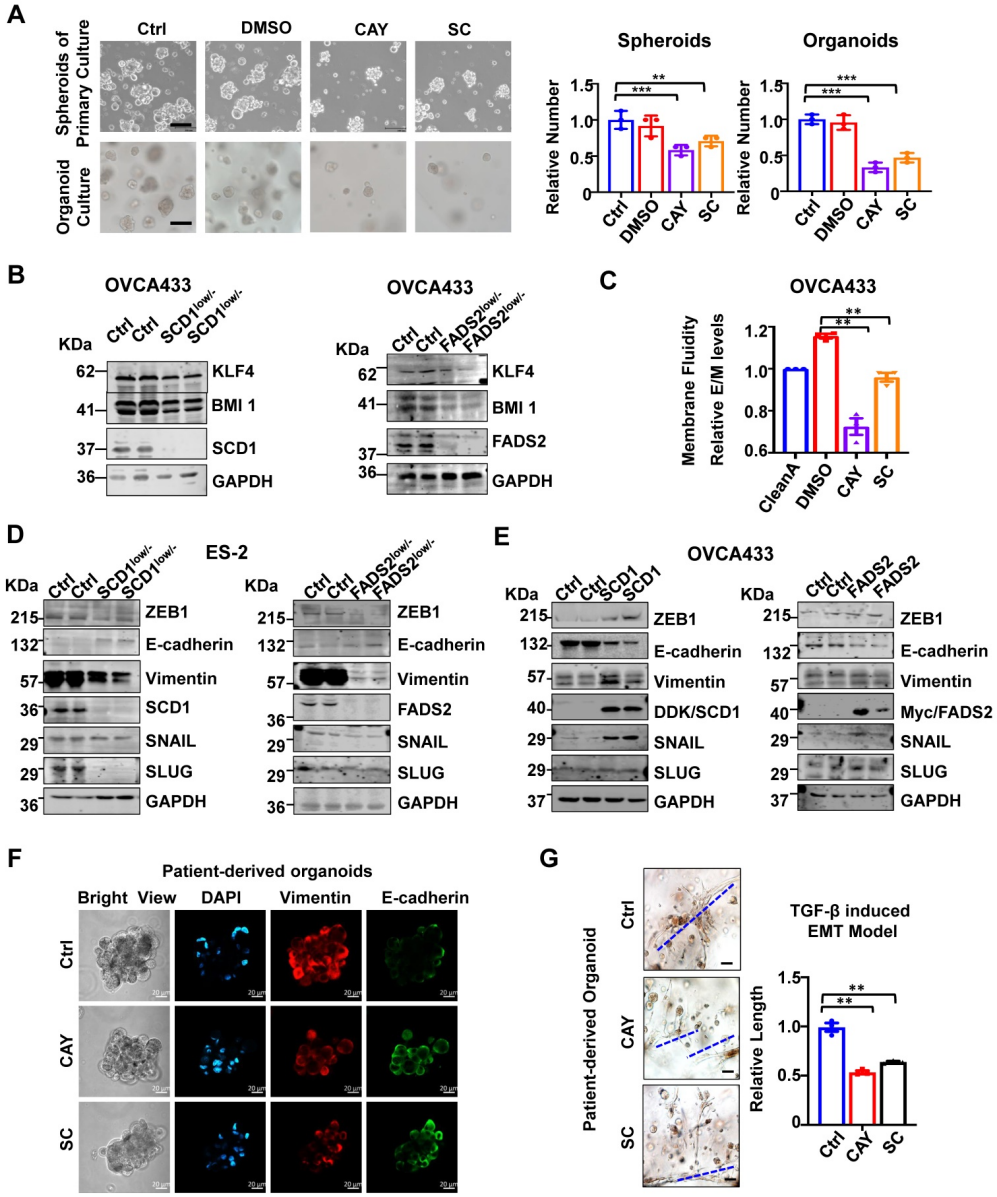
** SCD1/FADS2 deficiency attenuates stemness and correlates negatively with aggressive OvCa. (A)** Representative images and quantification of cell number in patient-derived spheroids and organoids after treatment with SCD1 inhibitor (CAY10566, 25 nM) or FADS2 inhibitor SC (sc26196, 500 nM) for 2 weeks. **(B)** Representative western blot analysis shows indicated proteins in SCD1^low/-^ or FADS2^low/-^ clones of OVCA433 cells. **(C)** Measurement of membrane fluidity evaluated by fluorescence spectroscopy quantification. OVCA433 were treated with SCD1 inhibitor (CAY10566, 10 nM) or FADS2 inhibitor (sc26196, 100 nM) for 48h. **(D and E)** Representative western blot analysis in **(D)** SCD1^low/-^ or FADS2^low/-^ clones of ES2 cells (same cells in Figure 2A) and **(E)** SCD1/FADS2-overexpressing clones of OVCA433 cells. **(F)** Representative fluorescence confocal images in OvCa patient-derived organoids. Scale bars, 100 µm. **(G)** TGF-β (5 ng/mL)-induced EMT model from patient-derived organoids treated with combined CAY10566 (25 nM) or sc26196 (500 nM) for 2 weeks. Scale bars, 100 µm. The results in **(A-G)** are representative findings of at least three independent experiments. Error bars represent the mean ± SEM. Statistical significance was determined by the two-tailed t-test. *P < 0.05, **P < 0.01, ***P < 0.001.

The spheroid formation ability of ascites-derived OvCa cells was suppressed by 32% and 29% upon treatment with SCD1 and FADS2 selective inhibitors, respectively (Figure 3A). As organoid self-renewal ability is another hallmark of stemness, treatment with SCD1 and FADS2 inhibitors led to reductions of 67% and 53% of the organoid self-renewal capability of OvCa cells, respectively (Figure 3A). Western blot analysis further revealed that stemness markers of KLF4 and BMI1 were downregulated by silencing of SCD1 or FADS2 in OVCA433 cells (Figure 3B). Compared with the negative controls (OCM pretreated with the lipid removal reagent, Cleanascite), OvCa cells co-cultured in the lipid-enriched OCM showed an increase of 18% in membrane fluidity. However, cotreatment with CAY10566 (10 nM) led to a 23% decrease in membrane fluidity, and cotreatment with sc26196 (100 nM) reduced the membrane fluidity to a level like that of the negative controls (Figure 3C). Collectively, these results suggest that activated SCD1/FADS2 is required for aggressive tumor initiative potential, and UFA-mediated membrane fluidity is crucial for maintaining OvCa cell stemness within the ascites microenvironment.

### Inhibition of SCD1/FADS2 impairs EMT transition in OvCa cells

Notably, silencing of SCD1 or FADS2 significantly suppressed mesenchymal markers (Vimentin) and EMT regulators (ZEB1, SNAIL, and SLUG) in ES2 SCD1^low/-^ and FADS2^low/-^ cells (Figure 3D). In contrast, enforced expression of SCD1 or FADS2 reversed the EMT markers in OVCA433 cells (Figure 3E). The data showed that Vimentin was downregulated, and E-cadherin expression was upregulated compared with the controls (Figure 3F). Gene Ontology (GO) and Kyoto Encyclopaedia of Genes and Genomes (KEGG) analyses of our transcriptomics on OVCA433 SCD1^low/-^ and FADS2^low/-^ cells supported that SCD1 and FADS2 participated in EMT-related biological processes and molecular functions (Figure S3A-D). In addition, upon treatment with CAY10566 (25 nM) or sc26196 (500 nM), the number and size of tumor organoids significantly decreased, and the spindle-like morphology was markedly disrupted by >1.89-fold compared with the controls (Figure 3G), suggesting that OvCa cells in EMT status were vulnerable to SCD1/FADS2 inhibition-based therapies.

### SCD1 and FADS2 are involved in protecting OvCa cells from oxidative stress

**Figure 4:**
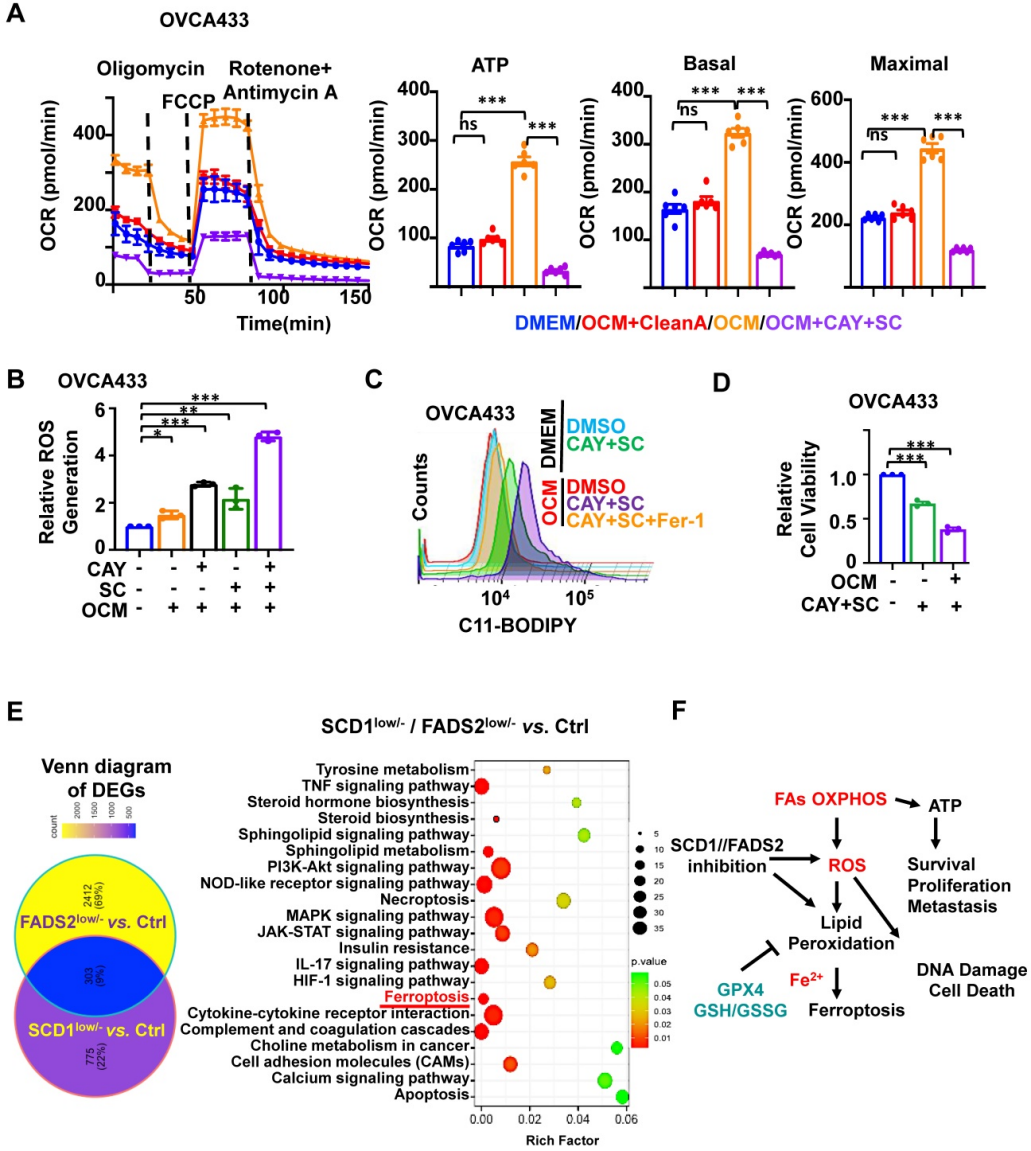
** SCD1/FADS2 blockade contributes to augmented cellular ROS and lipid peroxidation in metastatic OvCa. (A-D)** OCM-co-cultured ES-2 and OVCA433 cells were treated with the SCD1 inhibitor CAY (CAY10566, 10 nM), the FADS2 inhibitor SC (sc26196, 100 nM), or Fer-1 (Ferrostatin-1, 10 µM) for 48 h. **(A)** Measurement of the oxygen consumption rate (OCR). **(B)** Column scatter shows cellular ROS production. **(C)** Lipid peroxidation was measured by C11-BODIPY^581/591^ staining with flow cytometry. **(D)** Cell viability was measured by XTT assay. **(E)** Left: Venn diagram shows common and unique sets of differentially expressed genes among SCD1^low/-^, FADS2^low/-^, and control (Ctrl) OVCA433 cells. Right: KEGG analysis shows enriched signaling pathways in the common sets. **(F)** A schematic diagram shows the metabolic adaptation of FAO in metastatic OvCa cells. Both oxidant ROS and antioxidant GPX4 were highly expressed, and inhibition of SCD1/FADS2-mediated GPX4 suppression and elevated ROS/lipid peroxidation production promoted cell death.

To assess ATP and ROS production levels in OvCa cells, we utilized a Cell Mito Stress assay to measure ATP production. Of note, OCM-co-cultured OVCA433 cells exhibited 2.62-fold higher ATP production than DMEM (Figure 4A). In addition, ATP-linked basal OCR and maximal OCR revealed elevations of approximately 2.1- and 2.04-fold, respectively. Furthermore, the rescue experiment showed that OVCA433 cells co-cultured in lipid-depleted OCM lost the capacity for ATP production, indicating that the lipid-enriched microenvironment enhances the lipid metabolic activities of OvCa cells (Figure 4A). Similarly, the fluorescence intensity of H_2_DCFDA indicated that OCM-co-cultured OVCA433 cells exhibited a moderated elevation of ROS production compared with DMEM (Figure 4B).

We further confirmed SCD1/FADS2 is required to protect OvCa cells against ROS overproduction and ROS-stimulated cellular damage. Treatment of either CAY10566 (10 nM) or sc26196 (100 nM) in OVCA433 cells with OCM caused increased cellular ROS production by more than 1.5-fold compared to the DMEM negative and OCM positive controls. Notably, suppression of SCD1/FADS2 simultaneously induced a remarkable increase of more than 4.36-fold of ROS in the OCM co-culture system (Figure 4B). In contrast, we found that ATP production dramatically decreased, indicating that abrupt oxidative phosphorylation might be unable to meet the cellular ATP demands to support cell aggressiveness (Figure 4A). Our transcriptome profiling also supported this postulation that ROS-related pathways were significantly enriched in either SCD1- or FADS2-depleted clones, including Rho GTPases, MAPK signaling, ferroptosis, and other oxidative stress-related processes in OvCa cells (Figure S4A-B). Together, these findings propose that ascites-derived OvCa cells obtain high ATP production from the lipid-enriched microenvironment, while SCD1 and FADS2 not only exert fatty acid desaturase activity in producing UFAs but also equilibrate redox homeostasis to avoid ROS-mediated cell death.

Given that the inhibition of SCD1/FADS2 could elevate excessive intracellular ROS, it was hypothesized that the suppressed SCD1/FADS2 could induce lipid peroxidation in ascites-derived OvCa cells. As expected, the accumulation of lipid peroxidation was elevated by approximately 20% in OCM-co-cultured OVCA433 cells upon pharmacological inhibition of SCD1/FADS2 compared with the DMEM controls (Figure 4C). In the same co-cultured condition, mitochondrial lipid ROS was also increased (Figure S4C). Co-treatment of Fer-1 (10 μM) could prevent OVCA433 cells from producing excessive lipid peroxidation, cellular and mitochondrial ROS in OCM (Figure 4C and S4C). Notably, the cell viability was correspondingly reduced due to the excessive mitochondrial ROS and lipid peroxidation (Figure 4D). The findings demonstrated that SCD1/FADS2 inhibition-induced excessive ROS with lipid peroxidation is severely harmful to the survival of OvCa cells in the lipid-enriched microenvironment.

### Inhibition of SCD1/FADS2 promotes ferroptosis in ascites-derived OvCa cells

KEGG enrichment analysis of our transcriptional profiling revealed that the ferroptosis pathway was enriched as one of the critical signaling pathways after depletion of SCD1 or FADS2 in OvCa cells (Figure 4E). OvCa cells in the lipid-enriched microenvironment generate ATP via FAO along with cellular ROS production and mitochondrial ROS accumulation. It is thus hypothesized that a lipid-enriched microenvironment possibly enhances PUFA-based lipid peroxidation in OvCa cells when co-cultured with an SCD1/FADS2 inhibitor. Accordingly, abruptly elevated ROS might trigger apoptosis and ferroptosis to suppress the survival of OvCa cells, as summarized in Figure 4F. To distinguish whether apoptosis or ferroptosis is the main cause of cell death induced by SCD1/FADS2, the rescue experiment by leveraging the ferroptosis inhibitor Fer-1 (10 μM) and the pan-caspase inhibitor Z-VAD-FMK (10 μM) in reversing cell death was conducted in ES-2 cells co-cultured with OCM (Figure S4D-F). XTT cell viability assay demonstrated that the cell survival of ES-2 cells was remarkably higher upon the co-treatment of SCD1/FADS2 plus Fer-1 inhibitors than that of the co-treatment of SCD1/FADS2 plus Z-VAD-FMK inhibitors, indicating ferroptosis rather than apoptosis is more dominant in unleashing cell death (Figure S4F).

### GPX4 is the downstream target of SCD1/FADS2

**Figure 5:**
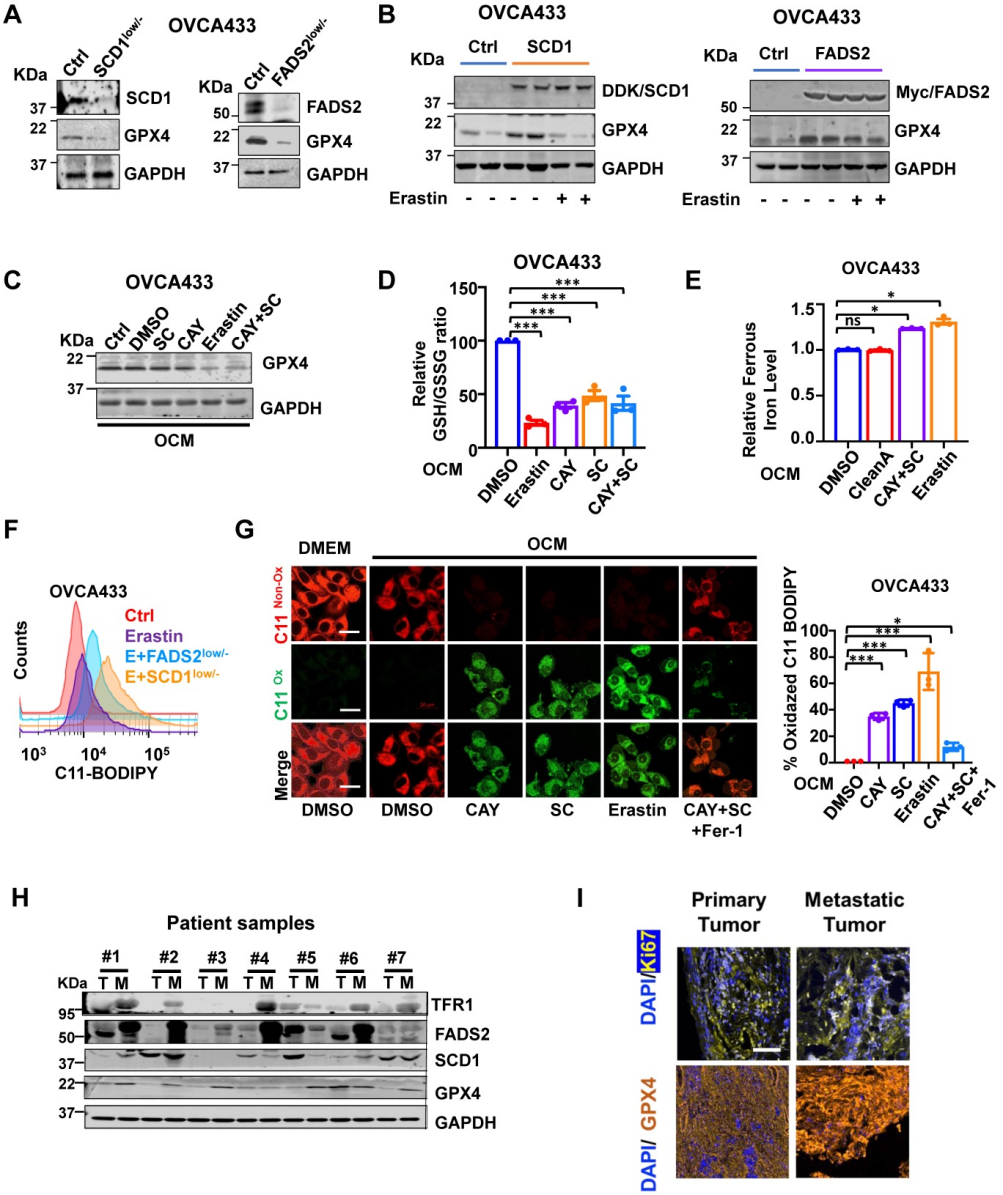
** Inhibition of SCD1/FADS2 increases cellular ROS and ferrous iron and downregulates GPX4 in OCM cotreated OvCa cells. (A and B)** Representative western blot analysis in SCD1/FADS2 **(A)** knockout clones and **(B)** stable overexpression clones of OVCA433. GAPDH was used as the internal control. **(C, D, E, G)** ES-2/OVCA433 cells co-cultured with OCM were treated with CAY10566 (10 nM), sc26196 (100 nM), Erastin (5 µM), or Ferrostatin-1 (10 µM) for 48 h. **(C)** Representative western blot. **(D)** Measurement of cellular GSH/GSSG ratio by fluorometric microplate. **(E)** Measurement of cellular ferrous. **(F)** Measurement of lipid peroxidation by C11-BODIPY^581/591^ staining with flow cytometry. **(G)** Left: Representative confocal images of lipid peroxidation. Red represents non-oxidation status, and the Green represents oxidation status. DMSO was used as the negative control (Ctrl). Scale bar, 20 µm. Right: Relative quantification of the percentage of green-positive lipid peroxidation. **(H)** Representative western blot analysis of indicated proteins in omental metastatic tumor tissues (M) and their primary counterpart tissues (T) in OvCa. GAPDH was used as the internal control. **(I)** Representative fluorescent mIHC images of 10 paired OvCa tissue samples. Scale bar, 100 µm. The results are representative of three independent experiments. For the results in **(D, F and G)**, error bars represent the mean ± SEM (*n* = 3). Statistical significance was determined by the two-tailed t-test. *P < 0.05, **P < 0.01, ***P < 0.001.

Based on our transcriptome profiling, Gene Set Enrichment Analysis (GSEA) analysis indicated that ferroptosis-related genes were extensively enriched in OVCA433 SCD1^low/-^ clones (*P* = 0.00086) (Figure S5A). Mechanistically, given the crucial role of GPX4 in ferroptosis, it is necessary to evaluate the expression level of GPX4 in relation to OvCa cells. To this end, western blot analysis showed that depletion of SCD1/FADS2 led to a decline in GPX4 expression in OVCA433 cells (Figure 5A). In contrast, forced expression of SCD1/FADS2 upregulated the level of GPX4 in OVCA433 cells (Figure 5B). The transcriptomic analysis confirmed these findings that the level of *GPX4* was significantly hampered in OVCA433 SCD1^low/-^ and FADS2^low/-^ cells, suggesting that SCD1/FADS2 are actively involved in the transcriptional activity of GPX4 (Figure S5B). Moreover, pharmaceutical inhibition of SCD1/FADS2, alone or in combination, was performed to examine the expression changes of GPX4. The results showed that the combined inhibition of SCD1 and FADS2 remarkably reduced the level of GPX4, and this reduction was equivalent to treatment with the ferroptosis inducer Erastin (5 μM) (Figure 5C). Using a glutathione assay, pharmaceutical inhibition of SCD1 and FADS2, individually or in combination, led to a reduction in the GSH/GSSG ratio by approximately 50% compared to the DMSO controls in ascites-derived OVCA433 cells (Figure 5D). These findings suggest that SCD1 or FADS2 deficiency attenuated GPX4 expression and activity in OvCa cells.

### Inhibition of SCD1/FADS2 elevates redox-active labile iron in OvCa cells

Numerous reports have shown that iron addiction promotes proliferation, evasion, and acquired chemoresistance in ovarian cancers 16-18. The aberrant upregulated iron-storage protein ferritin, and iron-containing enzymes could prevent iron-mediated lipid peroxidation and Fenton reaction 19. SCD1 and FADS2 are the key iron-containing enzymes, and mounting evidence has shown that the combined SCD1/FADS2 can bind iron at the center of their catalytic domain to execute enzymatic activities 20-22. Hence, the inhibition of SCD1/FADS2 could cause a lower iron-binding capacity leading to the increased cellular labile iron pool. As expected, iron quantification assays revealed that both Erastin (5 μM) and the combination treatment of CAY10566 (10 nM) and sc26196 (100 nM) in OVCA433 cells elevated the overall intracellular ferrous ion concentration (Fe^2+^) by 1.3-fold compared with either the DMSO negative control or Cleanascite-pretreated OCM (Figure 5E). This finding suggests that the suppression of SCD1/FADS2 activities led to the elevation of redox-active labile iron levels that, in turn, resulted in deregulated ROS accumulation and subsequent lipid peroxidation.

### Inhibition of SCD1/FADS2-induced ROS is associated with ferroptosis

The inhibition of SCD1/FADS2 downregulated GPX4 and elevated intracellular ROS simultaneously, it caused the level of lipid peroxidation was elevated by 20% in OCM-co-cultured OVCA433 cells upon cotreatment with Erastin and/or in combination with SCD1^low/-^/FADS2^low/-^ (Figure 5F). Consistently, the accumulation of lipid peroxidation was observed in OvCa cells upon pharmaceutical inhibition of SCD1/FADS2. The effect was equivalent to that of the ferroptosis inducer Erastin. Fer-1 rescued lipid peroxidation and led to a shift in fluorescence from green (oxidation) to red (nonoxidation) (Figure 5G). To investigate the mechanism of SCD1/FADS2 in preventing lipid peroxidation in metastatic OvCa cells, paired primary and metastatic ovarian tumor tissues were recruited. Western blot analysis showed that the expression levels of SCD1, FADS2, GPX4, and TFR1 in omental metastatic tumors were relatively higher than their counterpart primary tumor tissues (Figure 5H). Multiparametric IHC analysis also confirmed that GPX4 was highly expressed in omental metastatic tumor cells (Figure 5I). To further substantiate these findings, analysis of a publicly available dataset revealed that GPX4 was highly expressed in the advanced stage of OvCa, accompanied by high recurrence and platinum resistance (Figure S5C). Moreover, a positive correlation between SCD1/FADS2 and SCD1/GPX4 in OvCa tumor tissues was observed by regression analysis in TCGA-OV (Figure S5D). These data suggest that metastatic OvCa cells in the lipid-enriched microenvironment exhibiting high lipid metabolic activities to support their aggressive oncogenic properties and protect against ROS-mediated cell death or lipid peroxidation through the activated SCD1/FADS2/GPX4/TFR1 signaling axis.

### Cisplatin and SCD1/FADS2 inhibitors exhibit inapparent cell toxicity in noncancerous cells

Cisplatin is one of the first-line chemotherapeutic drugs for OvCa 23. The 50% inhibitory concentration (IC50) values of cisplatin in OVCA433 cells and ES-2 cells were 5.17 μM and 3.13 μM, respectively (Figure S6A), in line with other publications stating that a dose of cisplatin (0.5-5 μM) could abolish cancer cell growth 24, 25. Thus, a low dose of cisplatin (2 μM) was selected to perform the following combination treatment. Accordingly, a low dose of CAY10566 (5 nM) and sc26196 (100 nM) was chosen to perform the combinational treatment (Figure S6B). The XTT cell proliferation assay supported the combination of cisplatin with SCD1/FADS2 inhibitors exhibit unobvious cell toxicity in noncancerous cells (Figure S6C-E).

### SCD1/FADS2 inhibitors attenuate cisplatin resistance in OvCa cells

**Figure 6:**
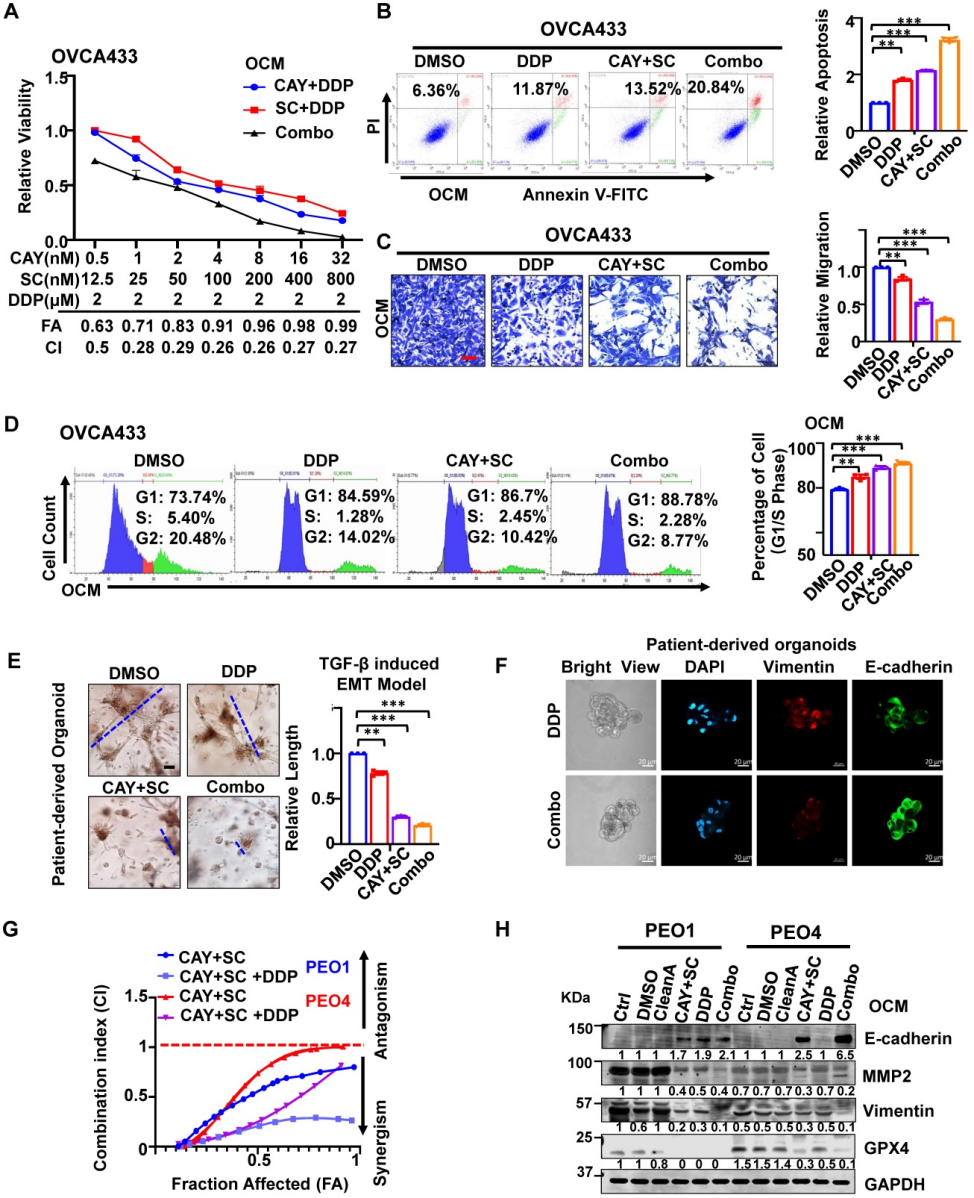
** Effects of SCD1/FADS2 inhibitors with or without cisplatin synergistically inhibit oncogenic and malignant transformation in OvCa and sensitizes OvCa to cisplatin. (A)** FA (fraction affected) and CI (combination index) values were calculated using the CalcuSyn software program.** (B-D, and H)** OCM-co-cultured OVCA433/PEO1/PEO4 cells treated with SCD1 inhibitor (CAY10566, 5 nM), FADS2 inhibitor (sc26196, 100 nM), DDP (cisplatin, 2 µM), combination (CAY10566+sc26196+cisplatin) or lipid removal reagent Cleanascite (CleanA) for 48 h. **(B)** Cell apoptosis was analyzed by flow cytometry. Cells were stained by Annexin V and propidium iodide (PI). **(C)** Representative images of the transwell migration assay. **(D)** Representative images of the cell cycle are determined by PI staining. **(E)** TGF-β (5 ng/mL)-induced EMT model from patient-derived organoids treated with combined CAY10566 (25 nM) and sc26196 (500 nM) for 2 weeks. Scale bars, 100 µm. **(F)** Representative fluorescence confocal images of patient-derived organoids show E-cadherin, Vimentin, and DAPI. Scale bars, 100 µm. **(G)** XTT results in PEO1 (cisplatin sensitive) and PEO4 (cisplatin-resistant) cells. FA (fraction affected) and CI (combination index) values were calculated using the CalcuSyn software program. **(H)** Representative western blot analysis. GAPDH was used as the internal control. The results are representative of three independent experiments. For the results in (**A-E**), error bars represent the mean ± SEM (*n* = 3). Statistical significance was determined by the two-tailed t-test. *P < 0.05, **P < 0.01, ***P < 0.001.

Upon co-treatment of CAY10566 (5 nM) or sc26196 (100 nM) with cisplatin (2 μM) in OVCA433 cells, cell viability was synergistically retarded in a dose-dependent manner with a combination index (CI) less than 1 (Figure 6A). Sole treatment with cisplatin (2 μM) and pharmacological inhibition of SCD1 and FADS2 in OVCA433 cells merely caused a 2-fold enhancement of the apoptotic rate (Figure 6B), whereas cotreatment of these inhibitors with cisplatin strengthened the apoptotic rate to 3.28-fold (Figure 6B). In addition, migration analysis demonstrated that the combined treatment of SCD1 and FADS2 inhibitors caused an approximately 2-fold reduction in cell migration, while a combination of cisplatin further reduced the migration of OVCA433 cells by 32% (Figure 6C). Treatment with SCD1/FADS2 inhibitors of OVCA433 cells led to G1/S cell cycle arrest compared with the controls (89.15%* vs.* 79.14%) (Figure 6D), while combinational treatment of these inhibitors with cisplatin generated substantial G1/S cell cycle arrest compared to the controls (91.06%* vs.* 79.14%) (Figure 6D). These findings suggest that cotreatment with SCD1/FADS2 inhibitors could synergistically inhibit cell growth and cell aggressiveness by promoting G1/S cell cycle arrest in OvCa cells. In addition to *in vitro* studies, we also provide a comprehensive preclinical evaluation of combining CAY10566, sc26196, and cisplatin in OvCa patient-derived organoids. Combination treatment with CAY10566 (25 nM) and sc26196 (500 nM), with or without cisplatin (2 μM), significantly disrupted the spindle-like morphology of the patient-derived organoids by more than 2.43-fold (Figure 6E). As apparent from the IF assay, Vimentin was downregulated, and E-cadherin was upregulated after combination treatment with CAY10566, sc26196, and cisplatin (Figure 6F), indicating that OvCa cells with a mesenchymal phenotype during malignant transformation are susceptible to this combination regimen.

An isogenic paired OvCa cell line, PEO1 (cisplatin-sensitive) and PEO4 (cisplatin-resistant), was chosen to identify this assumption. Combination treatment of PEO1 and PEO4 cells with CAY10566 (5 nM), sc26196 (100 nM), and cisplatin (2 μM) synergistically displayed the maximal inhibitory effect on cell viability with increasing values of fraction affected (Fa), and a combination index (CI) less than 1 (Figure 6G). Upon combination treatment of PEO1 and PEO4 cells, the expression of the mesenchymal marker Vimentin, the migration protein MMP2, and the ferroptosis marker GPX4 was severely decreased, whereas the expression of the epithelial marker E-cadherin was apparently elevated compared with either treatment with cisplatin or SCD1/FADS2 inhibitors (Figure 6H). Collectively, these data provide solid preclinical evidence supporting the potential clinical use of SCD1/FADS2 inhibitors in combination with cisplatin for eradicating OvCa metastasis.

### SCD1/FADS2 inhibitors and cisplatin synergistically enhance *in vivo* anticancer effect

**Figure 7:**
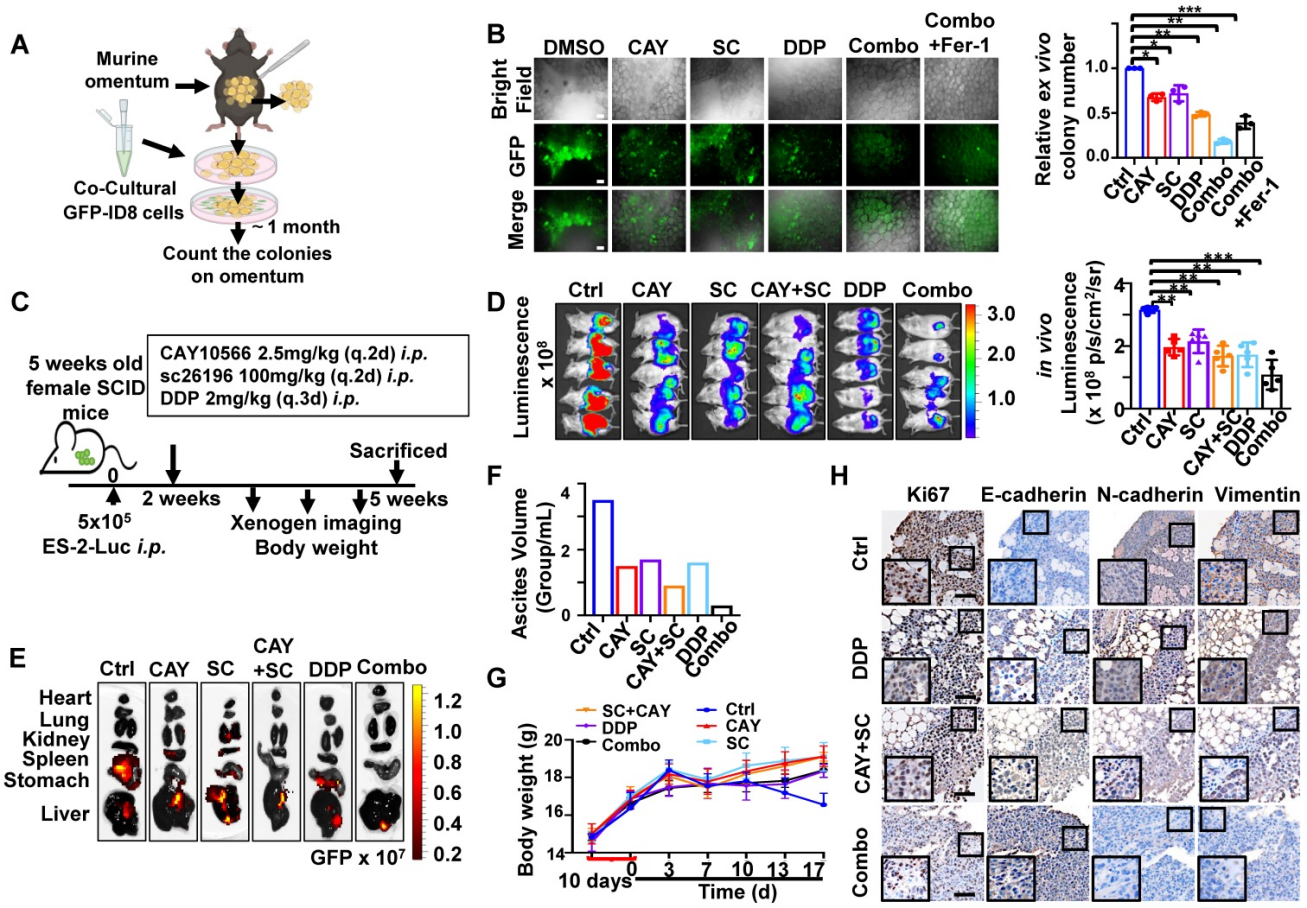
** SCD1 and FADS2 deficiency combined with cisplatin is highly efficacious in suppressing OvCa peritoneal metastases *in vivo.* (A)** Schematic diagram showing the *ex vivo* protocol of the omental metastasis model. The fresh omental tissue of C57BL/6 mice co-cultured with GFP-ID8 mouse OvCa cells with each treatment for 30 days. The treatments in each group were Ctrl (DMSO), CAY (CAY10566, 25 nM), SC (sc26196, 500 nM), DDP (cisplatin, 5 µg/mL), combination (CAY10566+sc26196+cisplatin), and combination+Fer-1 (combo+Ferrostatin-1). Fer-1 (20 µM) **(B)** Left: Representative images captured by ZOE Fluorescent Cell Imager show fluorescence of tumor colonization in the *ex vivo* model. Scale bar, 100 µm. Right: Quantification of tumor colonization numbers in an *ex vivo* model. Total viable colonies were measured. **(C)** The schematic diagram shows the experimental strategy for *in vivo* OvCa treatment. SCID mouse OvCa peritoneal metastases model was established by GFP-ES-2 cell intraperitoneal injection. After 2 weeks, the mice were subjected to different treatments. CAY10566 (2.5 mg/kg, q.2d), sc26196 (100 mg/kg, q.2d), and cisplatin (2 mg/kg, q.3d) were administered separately or in combination. **(D)** Luminescence IVIS images and quantitative analysis show peritoneal metastases after each treatment. **(E)** GFP fluorescence IVIS images show metastasis in different organs. **(F)** Average mouse ascites volume. **(G)** Average mouse body weight. **(H)** Representative IHC images show EMT markers in resected mouse tumors. Scale bar, 100 µm. The results are representative of three independent experiments. For the results in (**B, D, and G**), error bars represent the mean ± SEM (*n* = 3). Statistical significance was determined by the two-tailed t-test. *P < 0.05, **P < 0.01, ***P < 0.001.

To further evaluate the synergistic, therapeutic effect of combination drugs in OvCa progression and peritoneal metastases, we first identified them in a murine omentum culture *ex vivo* model (Figure 7A) 6. C57BL/6 mouse omental tissues with metastatic GFP-ID8 cell colonies were treated for 30 days with vehicle, CAY10566 (25 nM), sc26196 (500 nM), cisplatin (5 μg/mL), or a combination. According to the fluorescence microscopy results, in each treatment group compared with the vehicle group, the number of metastatic tumor colonization had a decrease of 43-59%, whereas the two inhibitors combined with cisplatin had a dramatic inhibition of 87%; in the rescue group, Fer-1 (20 μM) neutralized the efficacy of the three-drug combinations, and the colony number showed a decrease of only 75% (Figure 7B).

Next, we established an *in vivo* omental metastasis mouse model by intraperitoneal (*i.p.*) GFP and luciferase dual-labeled ES-2 cells (Figure S7A). The schematic diagram shows the experimental timeline of the treatment (Figure 7C). Peritoneal tumor-bearing SCID mice were treated with vehicle, CAY10566, sc26196, cisplatin, separately or in combination for two weeks. Notably, severe peritoneal metastases were solely observed in the control group (Figure 7D and S7B), the luminescence quantification value revealed a decrease of approximately 32.7% to 61.4% (Figure 7D), and the GFP fluorescence value exhibited a decrease of approximately 33.2% to 81.4% (Figure S7B). The control group mice showed widespread metastatic dissemination among the kidneys, spleen, stomach, and liver. In contrast, the three-drug combination group only showed small lesions of liver metastasis (Figure 7E). The control group mice had noticeable ascites, which was 2.33- fold more than the treatment groups. Notably, the mice treated with the combination of three drugs only had less than 0.5 mL of ascites in total (Figure 7F). The control group mice had a rapidly deteriorating body weight, whereas there was no significant change in body weight within the treatment groups, suggesting that all of the drugs were well tolerated *in vivo* (Figure 7G). Histopathologic examination revealed no significant injury to mouse organs after a long therapy duration (Figure S7C). The IHC results confirmed that the combination of three drugs significantly suppressed EMT (Figure 7H). These results indicate that clinically applicable cisplatin synergizes with CAY10566 and sc26196 to suppress OvCa peritoneal metastases *in vivo*.

## Discussion

In this study, we report that SCD1 and FADS2 are two dominant fatty acid desaturases (FADs) involved in *de novo* synthesis of PUFAs and MUFAs that, in turn, augment the capacities of cell growth, membrane fluidity, CSC formation, and EMT in OvCa cells derived from the ascites microenvironment. Given that high lipid metabolic activities support OvCa oncogenic properties, it is conceivable to find high ROS production in ascites-derived OvCa cells. Intriguingly, our study revealed that SCD1/FADS2 could modulate the GSH/GSSG ratio and GPX4 to avoid excess ROS in mediating oxidative stress and even ferroptosis. Notably, combined inhibition of SCD1/FADS2 triggered ferroptotic cell death. Importantly, harnessing commercially available inhibitors to selectively suppress SCD1/FADS2 activities could mediate the synergistic anticancer effect of cisplatin, providing novel and alternative therapeutic regimens for combating OvCa chemoresistance and peritoneal metastases.

Our recent study found that lipid-enriched ascites enforces OvCa cells to undergo metabolic reprogramming and utilize FAs as major energy sources for aggression and development 6. Intriguingly, SCD1 and FADS2 have been mentioned to modulate UFA synthesis 8 and participate in different oncogenic signaling pathways to promote tumor proliferation, migration, and stemness in transcription and posttranscriptional ways 26, 27. SCD1/FADS2-catalyzed formation of oleic acid (OA) and arachidonic acid (AA) appear to be the major substrate for bioactive lipids that promote cancer cell oncogenic properties including proliferation, migration, and stemness 28, 29. In line with this piece of evidence, lipidomic profiling analysis in this study revealed that the aberrant upregulation of SCD1/FADS2 desaturation activities could elevate *de novo* synthesized UFAs and β-oxidation activities in OvCa cells derived from ascites or OCM co-cultures. Notably, the upregulation of SCD1/FADS2 was commonly observed in metastatic tumor tissues compared to primary tumor tissues in EOC, indicating their clinical significance. *De novo* FA synthesis and metabolic enzymes are usually inactivated in normal cells, whereas the increased activities of *de novo* FA synthesis and FAO are the primary activities supporting the rapid growth and metastatic potential of cancer cells 30, 31. On the other hand, the high energy demand in cancer cells usually causes excessive ROS production, and the antioxidant defence system is simultaneously activated to prevent cell damage 32. Tesfay *et al*. showed that SCD1 could protect HGSOC cells from excessive oxidative damage through modulating MUFAs composition 33. However, in the ascites-derived metastatic OvCa cells, both MUFAs and PUFAs constituted the elevated UFAs due to the activities of SCD1 and FADS2. Indeed, studies have recently found that intracellular excess PUFAs promote membrane phospholipid (PUFA-PL) formation, increase membrane thickness and elevate lipid droplet storage 34, 35. PUFAs (especially C22:4 and C20:4), instead of MUFAs, are the most favorable substrate for cellular oxidants to attack lipids containing carbon-carbon double bonds 34. Metastatic OvCa cells are characterized by a higher PUFAs content in membrane and copious ROS production as compared with normal cells 36. The altered lipid metabolism and, subsequently, the abnormal high PUFA membrane is one of the most significant properties of metastatic cancer cells 37, 38. Consistently, our findings showed that the high PUFAs lead to increased membrane fluidity which is crucial for maintaining OvCa cell stemness, metastatic properties, and chemoresistance. Overall, the abundant membrane phospholipids and ROS generation during rapid fatty acid (FA) β-oxidation render OvCa cells vulnerable to therapeutic strategies that could interrupt redox homeostasis, thereby opening opportunities for precision remedies via aiming novel druggable targets such as SCD1/FADS2 37, 39. Indeed, the selective inhibitors of SCD1 (CAY10566) and FADS2 (sc26196) have no serious side effects 8, 40, 41, suggesting both inhibitors have great potential for clinical use.

Cell fate decision in response to SCD1/FADS2 inhibition is determined by multiple levels such as cell-cycle arrest, apoptosis, and ferroptosis 33, 42, but there is still no comprehensive study to compare their relative therapeutic contributions. DNA damage and nutrient deprivation are the two major causes of cell cycle arrest 43. During the cell cycle process of mitosis, SCD1 and FADS2 are required for stimulating lipid biosynthesis to supply new phospholipids for cell membrane biogenesis. This study demonstrated that CRISPR-mediated gene silencing or pharmacological inhibition of SCD1/FADS2 caused G1/S cell cycle arrest in OvCa cells. However, there was no significant difference in G1/S cell cycle arrest levels of OvCa cells co-cultured in either DMEM or OCM. The possible explanation is that the ascites microenvironment contains plenty of free fatty acids (FFAs), providing the source for membrane biogenesis. In contrast, inhibition of SCD1/FADS2 induced a higher reduction of the cell viability in OvCa cells co-cultured in OCM. Of note, the significant reduction of OvCa cell survival was inversely correlated with the increased cellular/mitochondrial ROS production and lipid peroxidation upon treatment of SCD1/FADS2 inhibitors in lipid enriched microenvironment. Intriguingly, by using the rescue experiment, we demonstrated that ferroptosis, instead of cell apoptosis, is the dominant event in unleashing cell death of OvCa cells induced by SCD1/FADS2 inhibition in the lipid-enriched TME.

Ferroptosis is a novel oxidative form of cell death associated with increased lipid peroxidation through the accumulation of lipid peroxides in an iron-dependent manner 44, 45. Given that the ascites-derived OvCa cells exhibit high lipid metabolic activities with elevated PUFAs content in membrane lipids, it is of interest to examine the mechanistic role of SCD1/FADS2 in ferroptosis. Previous studies have already reported that OvCa relies on iron (Fe^2+^) for proliferation and evasion, and a simultaneous increase in ROS production could result in ferroptosis 46. However, the associated mechanisms remain unclear. Tesfay *et al*. has shown that inhibition of SCD1 caused the reduction of coenzyme Q10 (CoQ10), which is a potent anti-oxidant preventing in mitochondrial dysfunction, and subsequently induces ferroptosis in OvCa cells 33, 47. This study reports a novel cellular antioxidant system that allows metastatic OvCa cells in the lipid-enriched microenvironment to adapt to ROS insult by upregulating SCD1, FADS2, and GPX4 in OvCa cells to avoid oxidative stress. Our transcriptome profiling indicated that the depletion of SCD1 or FADS2 in OvCa cells led to the enrichment of ROS-related signaling pathways. In addition, our mechanistic studies found that SCD1/FADS2 could prevent ROS-mediated oxidative stress by positively regulating the antioxidant GPX4 and GSH/GSSG ratio in OvCa cells co-cultured in the lipid-enriched microenvironment. GPX4 is widely localized in the extracellular, plasma membrane, and organelle membrane 48. Targeted suppression of GPX4 causes lipid peroxidation and thus induces ferroptotic cell death 49. Our study identified SCD1/FADS2 inhibition could positively regulate GPX4 in translation and transcription levels. This indicates GPX4 is the key antioxidant in preventing lipid peroxidation in the membrane of ascites-derived OvCa cells, while SCD1/FADS2 are the positive regulators of GPX4. This observation reinforces the critical roles of SCD1/FADS2 in promoting OvCa development and progression. Strikingly, cellular redox balancing was significantly abrupt after combined treatment with SCD1 and FADS2 inhibitors. Increased cellular lipid peroxidation is mainly found in ascites-derived OvCa cells because of the sufficient imbibed PUFAs at the membrane, creating susceptible lipid peroxidation when the antioxidant system is interrupted by SCD1/FADS2 inhibitors. Other studies have supported our findings that inhibition of SCD1/FADS2 could induce cellular ROS by activating typical oxidative stress response pathways including terminal UPR signaling and IRE1/JNK signaling, and abrogate NRF2/Keap1 antioxidant activity in several cancers 50, 51. Collectively, this study suggests that harnessing inhibitors of SCD1 and FADS2 to selectively remove ascites-derived cancer cells could be a promising chemotherapeutic regimen to eradicate peritoneal metastases of OvCa and exhibited no cytotoxicity in normal cells and *in vivo*. According to molecular and clinical studies, several reasons have been suggested 10, 52. For instance, GSH acts as a cofactor in facilitating MRP2-mediated cisplatin efflux to induce cisplatin resistance in cancer chemotherapy 53. In addition, ABC transporters function as efflux pumps to eliminate antitumor drugs, such as cisplatin 54. In this study, the combination of CAY10566, sc26196, and cisplatin efficiently inhibited cell viability and migration ability by inducing G1/S cell cycle arrest, apoptosis, and ferroptosis in OCM-co-cultured OvCa cells as the three-drug combination could suppress GPX4 and MMP2 expression in cisplatin-resistant PEO4 cells. Ferroptosis inducers in combination with cisplatin might elicit synergistic anticancer effects 55.

## Conclusions

In conclusion, metastatic OvCa cells have activated SCD1/FADS2 desaturation abilities and higher ROS production status than noncancerous cells. We herein take advantage of inhibition of SCD1 and FADS2 being able to synergistically abrupt ROS production to induce ferroptosis, sensitize cells to cisplatin-mediated cytotoxicity *in vitro*, and induce EMT suppression in an *ex vivo* culture of OvCa patient-derived organoids and *in vivo* peritoneal metastases animal study. Thus, targeting the suppression of SCD1/FADS2 combined with cisplatin seems to be a promising novel therapeutic strategy for improving treatment efficacy and against metastasis.

## Supplementary Material

Supplementary figures and table.

## Abbreviations

EOC: Epithelial Ovarian Cancer
OvCa: Ovarian Cancer
OCM: Omental Conditioned Medium
SCD1: Stearoyl-CoA desaturase-1
FADS2: Acyl-CoA 6-desaturase
UPLC-MS/MS: Ultra-performance liquid chromatography coupled to tandem mass spectrometry
SFA: Saturated Fatty Acid
UFA: Unsaturated Fatty Acid
MUFA: Mono-unsaturated Fatty Acid
PUFA: Poly-unsaturated Fatty Acid
POA: Palmitoleic acid
OA: Oleic acid
LOA: Linoleic acid
α-LOA: alpha-linolenic
γ-LOA: gamma-linolenic
AA: Arachidonic acid
TCGA: Cancer Genome Atlas
HOSEs: Human immortalized epithelial ovarian cells
FA: Fraction Affected
CI: Combination Index
CSC: Cancer Stem Cell
FAO: Fatty Acid β-oxidation
Fer-1: Ferrostatin-1
DDP: Cisplatin
Combo: Combination
