# SCD1/FADS2 fatty acid desaturases equipoise lipid metabolic activity and redox-driven ferroptosis in ascites-derived ovarian cancer cells: Erratum

**DOI:** 10.7150/thno.134905

**Published:** 2026-05-07

**Authors:** Yang Xuan, Huogang Wang, Mingo MH Yung, Fushun Chen, Wai-Sun Chan, Yau-Sang Chan, Stephen KW Tsui, Hextan YS Ngan, Karen KL Chan, David W Chan

**Affiliations:** 1 Department of Obstetrics & Gynaecology, LKS Faculty of Medicine, The University of Hong Kong, Hong Kong SAR, P.R. China.; 2 School of Biomedical Sciences, The Chinese University of Hong Kong, Hong Kong SAR, People's Republic of China.

The authors regret that in the original version of our paper, the Western blots for SCD1/FADS2-knockout ES2 clones in Figure 2A were duplicated with those in Figure 3D. They have replaced Figure 2A with independent Western blots from the same SCD1/FADS2 CRISPR/Cas9-edited ES2 clones, which validate the results presented in Figure 3D. This correction does not affect the original data or conclusions. The authors apologize for any inconvenience caused by this error.

## Figures and Tables

**Figure A:**
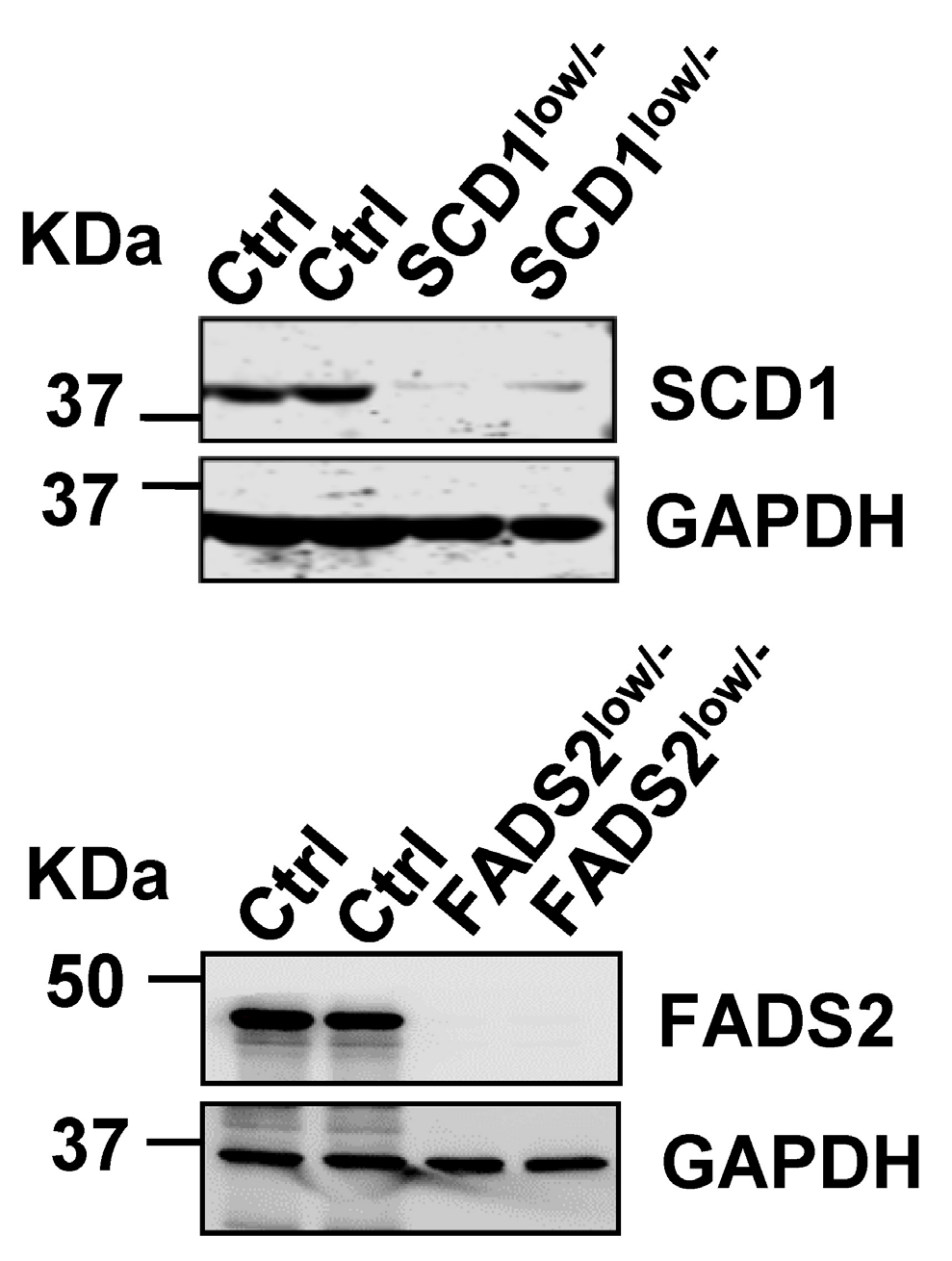
Corrected Figure 2A.

