# Supplementary material for: SCD1/FADS2 fatty acid desaturases equipoise lipid metabolic activity and redox-driven ferroptosis in ascites-derived ovarian cancer cells
Source: Theranostics. 2022 Apr 24;12(7):3534–52. doi: 10.7150/thno.70194 (PMC9065188; doi:10.7150/thno.70194)
Supplement: Supplementary file 1 — Supplementary figures and table. [file thnov12p3534s1.pdf]

Supplementary Materials:

SCD1/FADS2 fatty acid desaturases equipoise lipid metabolic activity and redox-driven ferroptosis in ascites-derived ovarian cancer cells

Yang Xuan[1], Huogang Wang[1], Mingo MH Yung[1], Fushun Chen[1], Wai-Sun Chan[1], Yau-Sang Chan[1], Stephen KW Tsui[2], Hextan YS Ngan[1], Karen KL Chan[1*] and David W Chan[1,2*]

[1] Department of Obstetrics & Gynaecology, LKS Faculty of Medicine, The University of Hong Kong, Hong Kong SAR, P.R. China
[2] School of Biomedical Sciences, The Chinese University of Hong Kong, Hong Kong SAR, People's Republic of China.

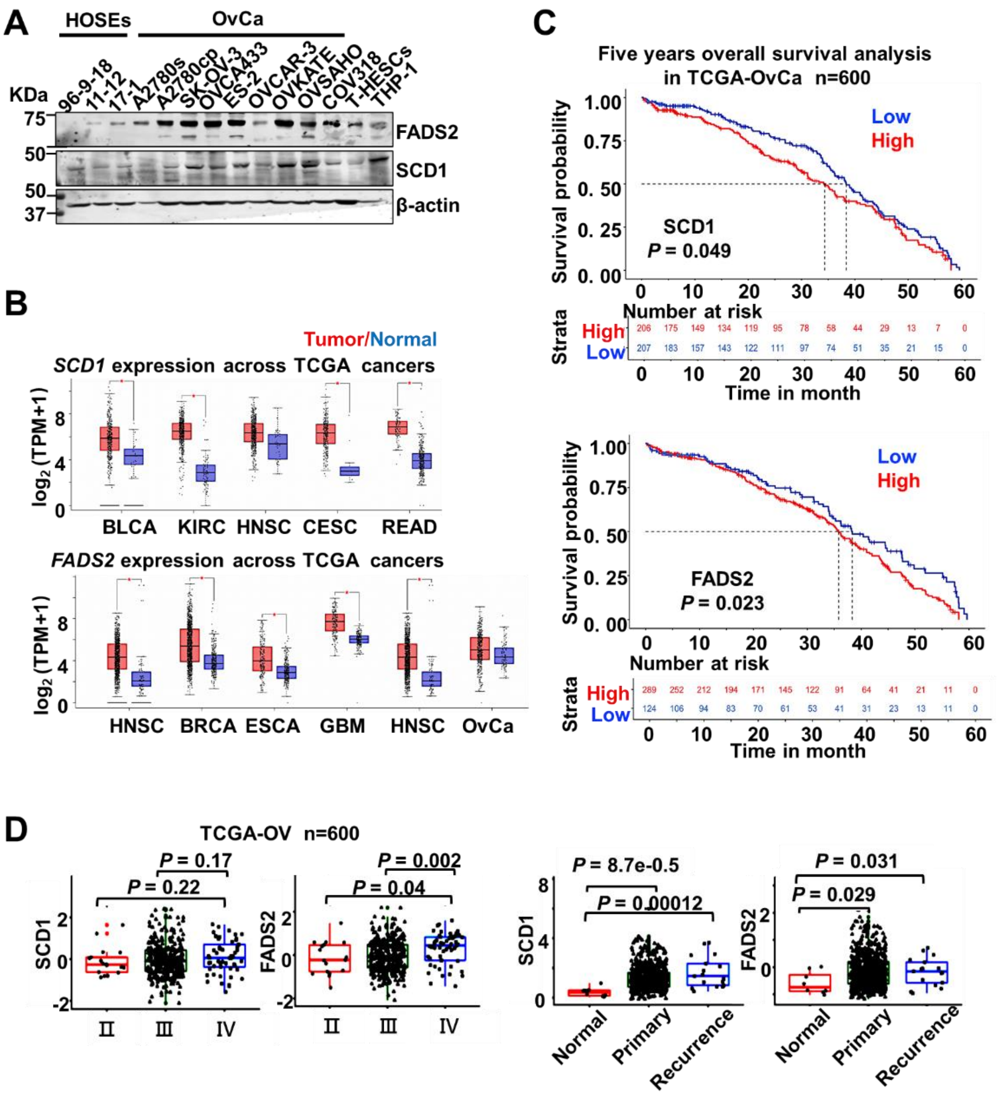

Figure S1. High expression of SCD1/FADS2 is a poor prognostic indicator for OvCa. Related to Figure 1

(A) Representative western blot analysis of SCD1 and FADS2 highly expressed in OvCa cell lines compared to HOSEs and negative control cell lines; T-HESCs (Stromal Fibroblasts) and THP-1 (Monocytes). β-actin was used as the internal control. The results are three independent experiments with similar results.
(B) Comparison of *SCD1/FADS2* mRNA expression in multiple clinical tumor samples, including BLCA (Bladder Carcinoma), KIRC (Kidney Carcinoma), HNSC (Head and Neck Carcinoma), CESC (Cervical Cancer), READ (Rectum Adenocarcinoma), BRCA (Breast Carcinoma), ESCA (Esophageal carcinoma), GBM (Glioblastoma), OvCa (Ovarian cancer). Data were retrieved from The Genotype-Tissue Expression (GTEx) project from the TCGA portal. Statistical significance was determined. *$P < 0.05$
(C and D) TCGA-OvCa database (assession number: phs000178, n=600) was analyzed **(C)** Kaplan-Meier survival analysis of indicated groups. Red represents high expression, blue represents low expression; and **(D)** the mRNA expression of SCD1 or FADS2 in different tumor stages (II, III, IV), normal ovary tissues (normal), primary tumor tissues (primary), and recurrence tissues (recurrence). *P*-value was calculated by a two-tailed t-test in R studio.

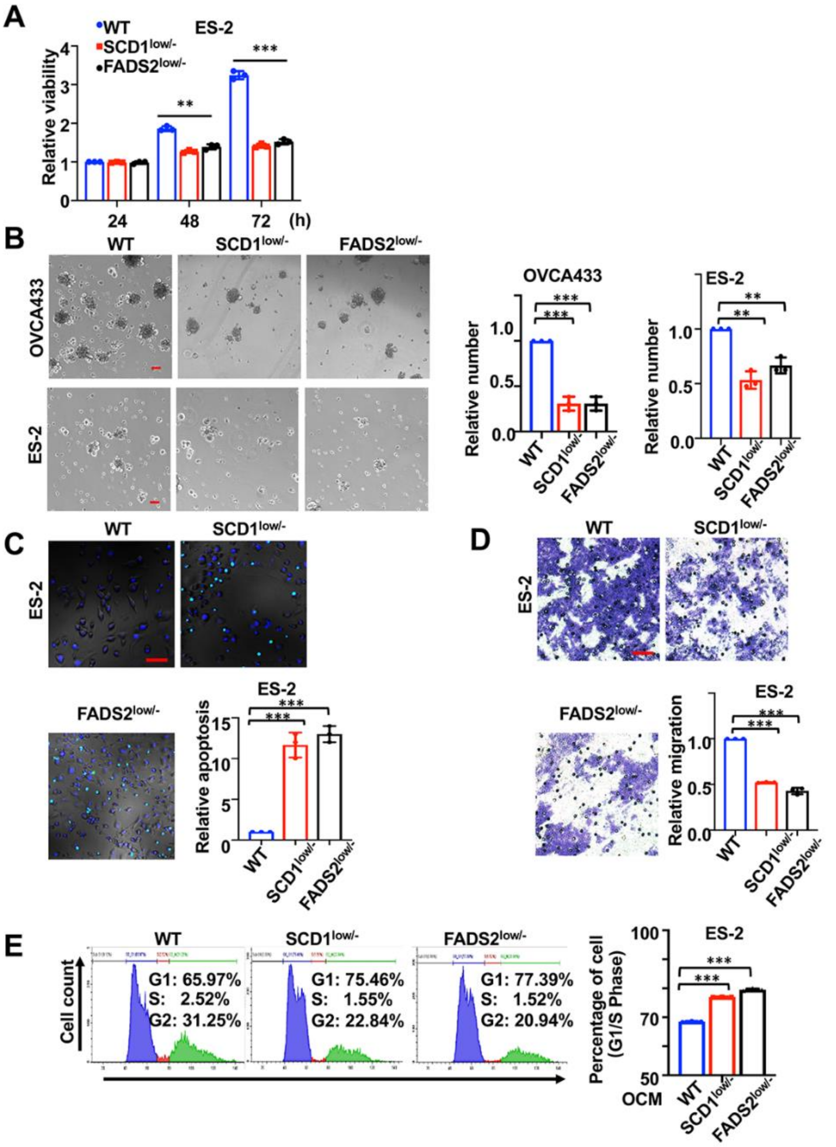

Figure S2. SCD1/FADS2 silencing suppresses the oncogenic role in OvCa cells. Related to Figure 2

(A) XTT cell proliferation analysis shows decreased cell viability in SCD1$^{low/-}$ or FADS2$^{low/-}$ clones of ES-2 cells.

(B) Represented images of tumor-sphere formation in SCD1$^{low/-}$ or FADS2$^{low/-}$ clones of OVCA433 and ES-2 cells.

(C) Represented caspase3/7 fluorescent images show increased apoptosis in SCD1$^{low/-}$ or FADS2$^{low/-}$ clones of ES-2 cells. Nuclei were stained by Hoechst (Blue), caspase3/7 positive (Green).

(D) Represented images of Transwell migration assay show decreased migration ability in SCD1$^{low/-}$ or FADS2$^{low/-}$ clones of ES-2 cells.

(E) Represented images of cell cycle determined by PI staining show G1/S arrest in SCD1$^{low/-}$ or FADS2$^{low/-}$ clones of ES-2 cells.

Results in **(A-E)**, SCD1$^{low/-}$ and FADS2$^{low/-}$ were compared to control wild type (WT). Quantification was mean ± SEM (n=3 independent experiment). Statistical significance was determined by a two-tailed *t*-test. *$P < 0.05$, **$P < 0.01$, ***$P < 0.001$.

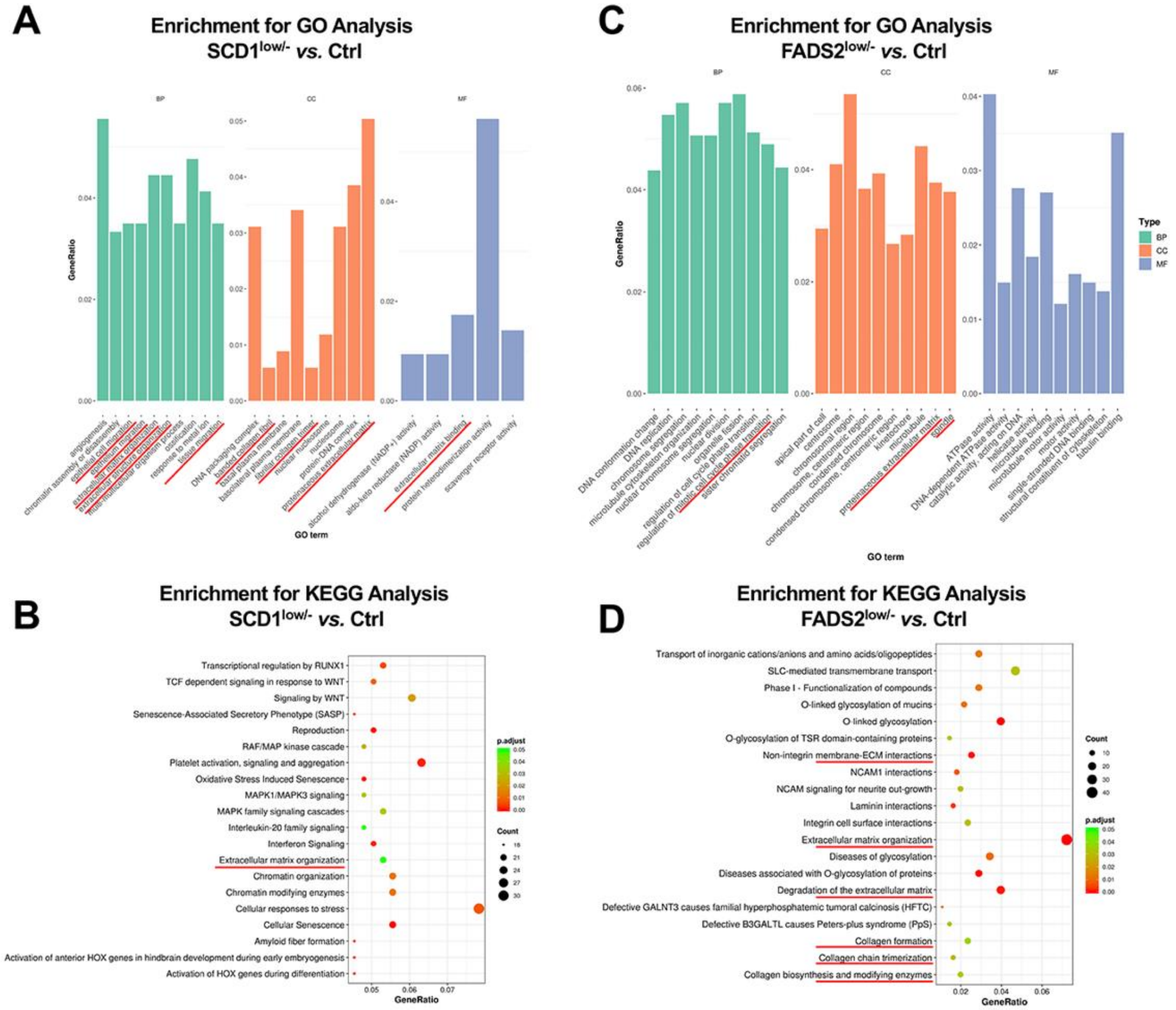

Figure S3. SCD1/FADS2 silencing enriched EMT-related biology processes in OvCa cells. Related to Figure 3

(A, B) RNA-seq GO analysis and KEGG analysis show ECM-related biology processes in SCD1$^{low/-}$ clones of OVCA433 cells.
(C, D) RNA-seq GO analysis and KEGG analysis show ECM-related biology processes in FADS2$^{low/-}$ clones of OVCA433 cells.

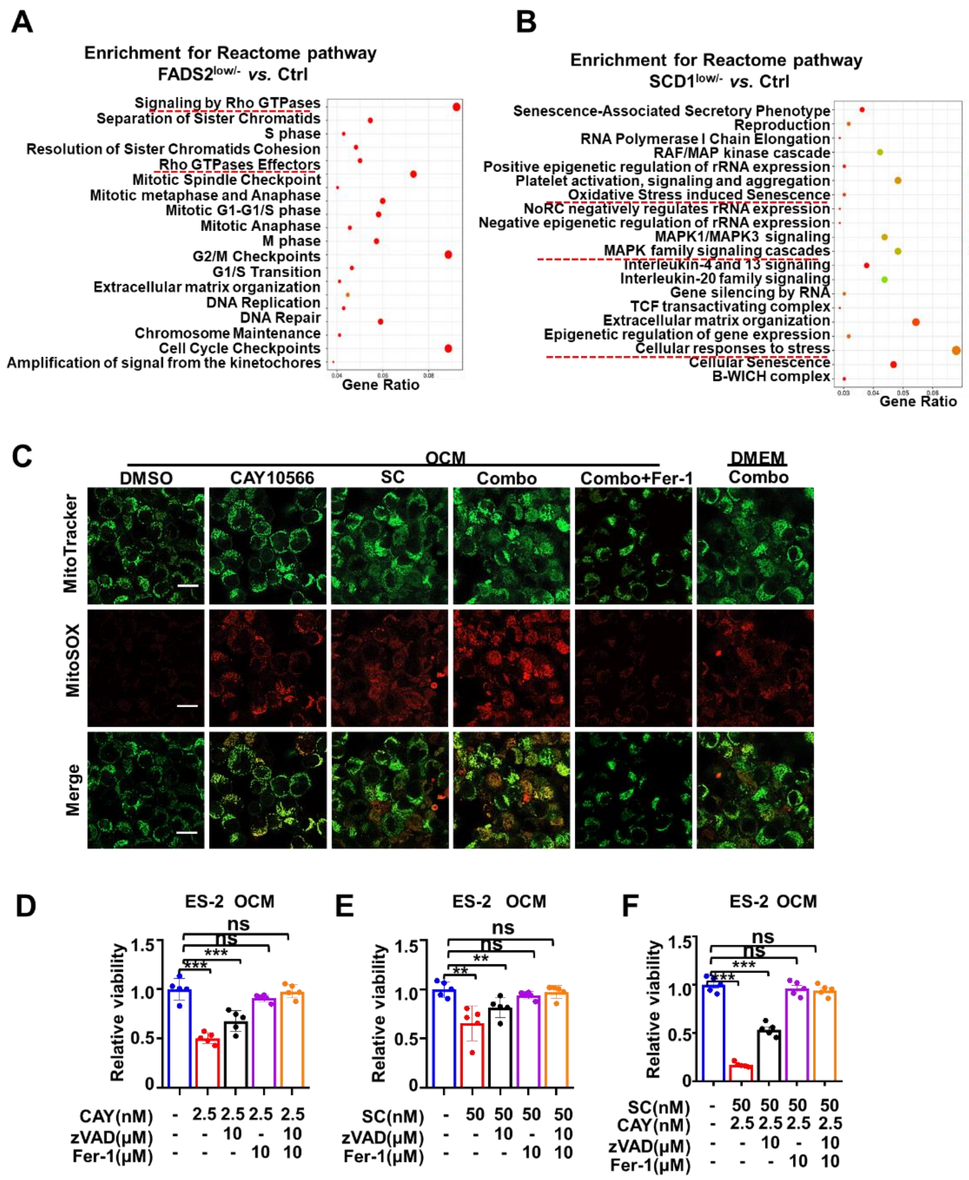

Figure S4. Silencing of SCD1/FADS2 promotes oxidative stress-related signaling pathways in OvCa cells.

Related to Figure 4

(A) RNA-seq Reactome Pathway analysis shows oxidative stress-related pathway RHO GTPases activated in FADS2$^{low/-}$ clones of OVCA433 cells.

(B) RNA-seq Reactome Pathway analysis shows oxidative stress-related pathways activated in SCD1$^{low/-}$ clones of OVCA433 cells.

(C) Representative fluorescence confocal images of mitochondrial ROS cells were stained by a MitoSOX probe. MitoTracker dye is the mitochondrial marker, Scale bar, 20 µm. OCM or DMEM cocultured OVCA433 cells treated with SCD1 inhibitor (CAY10566, 5 nM), FADS2 inhibitor (sc26196, 100 nM), lipid ROS scavenger (Fer-1, 10 µM), or combination (CAY10566+sc26196) for 24 h. **(D-F)** Cells were treated with SCD1 inhibitor CAY (CAY10566), FADS2 inhibitor SC (sc26196), apoptosis inhibitor (Z-VAD-FMK), ferroptosis inhibitor (Fer-1) for 48h. XTT assay detected cell viability after each treatment in OCM co-cultured ES2 cells. Results in **(D-F)** are mean ± SEM (n = 3). Statistical significance was determined by a two-tailed t-test. ns > 0.05.

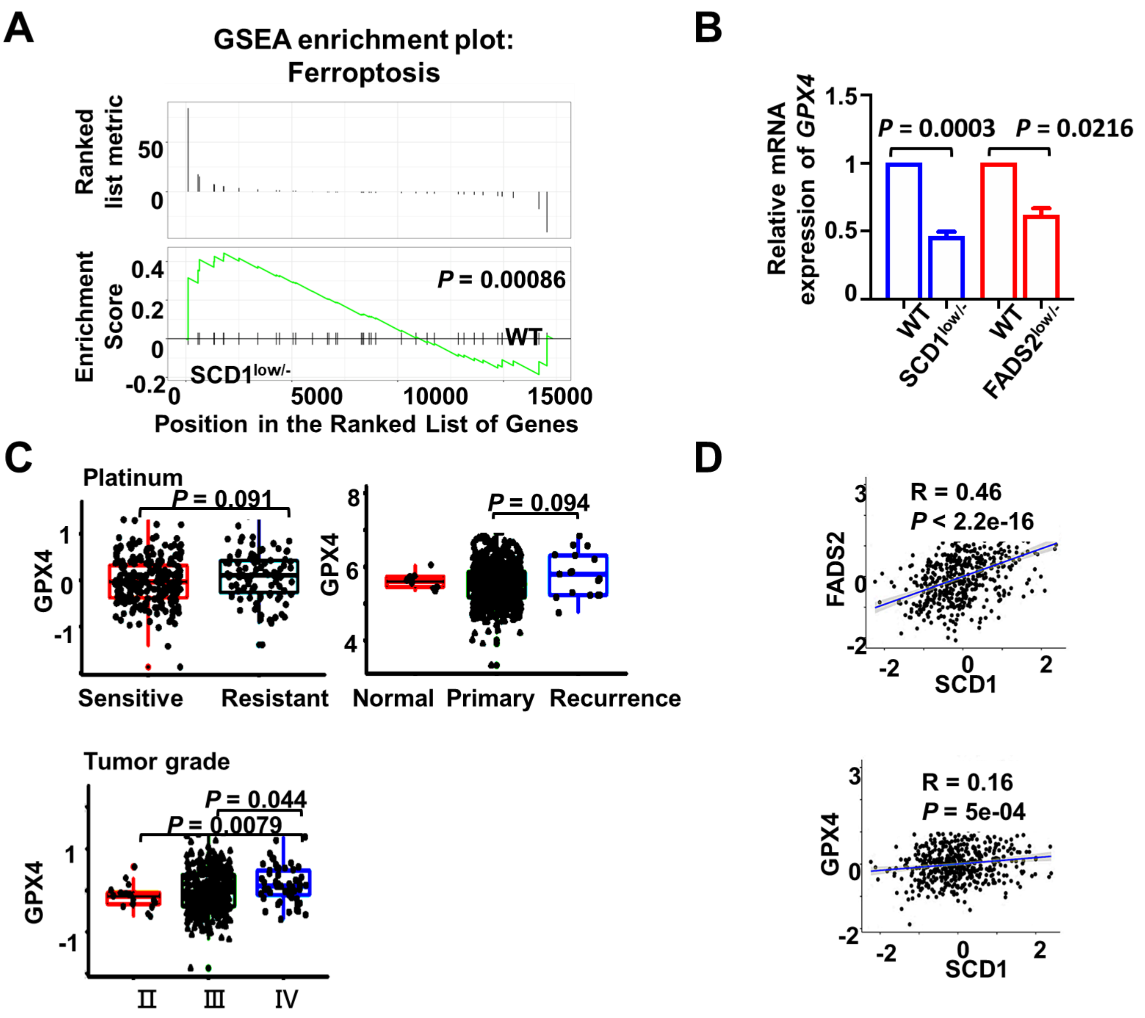

Figure S5. Silencing SCD1/FADS2 enriches the ferroptosis signaling pathway, and SCD1/FADS2 are positively correlated with GPX4 in OvCa.
Related to Figure 5

(A) KEGG analysis shows the ferroptosis pathway is enriched in SCD1$^{low/-}$ clones of OVCA433 cells ($P = 0.00086$).

(B) Bar chart shows fold changes of ferroptosis-related genes mRNA expression in SCD1$^{low/-}$ clones of OVCA433 cells.

(C and D) TCGA-OV database (assession number: phs000178, n = 600) was analyzed. **(C)** The mRNA expression of GPX4 and xCT in different tumor stages (II, III, IV), normal ovary tissues (normal), primary tumor tissues (primary), recurrence tissues (recurrence), platinum-sensitive, and -resistance statue. *P*-value was calculated by a two-tailed *t*-test in R studio.

(D) The positive correlation between SCD1/FADS2 and GPX4 expression was detected. The Pearson's correlation *P*-value was calculated in R studio.

A

IC50(μM)	DDP
OVCA433	5.17
ES-2	3.13

B

IC50(nM)	CAY10566	sc26196
OVCA433	11.91	243.47
ES-2	7.37	117.89
HOSE11-12	21.74	373.98
T-HESCs	12.89	358.13

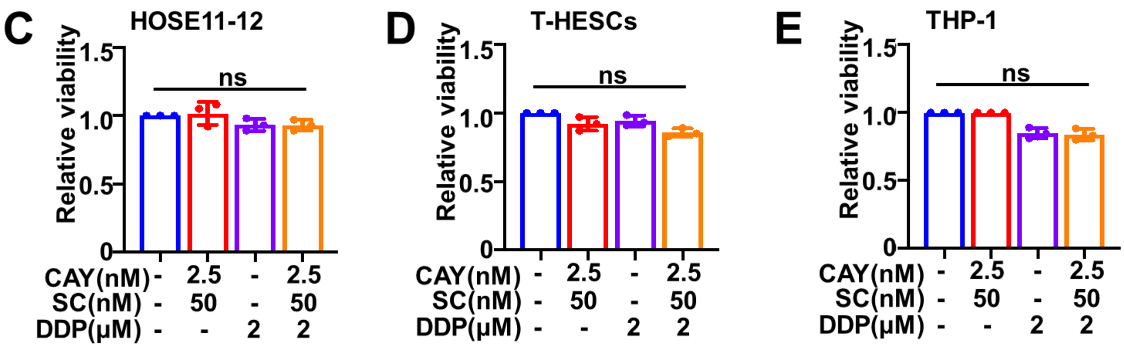

Figure S6. A combination of cisplatin with inhibitors of SCD1 and FADS2 has no toxicity to normal cells.
Related to Figure 6

(A) IC50 values of cisplatin for OVCA433, ES-2 cells.
(B) IC50 values of CAY10566, sc26196, and cisplatin for OVCA433, ES-2, HOSE11-12, and T HESCs cells.
Cells treated with SCD1 inhibitor CAY (CAY10566), FADS2 inhibitor SC (sc26196), DDP (Cisplatin) for 48h in **(C-E).**
XTT assay detected cell viability after each treatment in **(C)** normal ovarian HOSE11-12 cells, in **(D)** fibroblast T-HESCs cells, and in **(E)** macrophage THP 1 cells.
Results in **(C-E)** are mean ± SEM (n = 3). Statistical significance was determined by a two-tailed t-test. ns > 0.05.

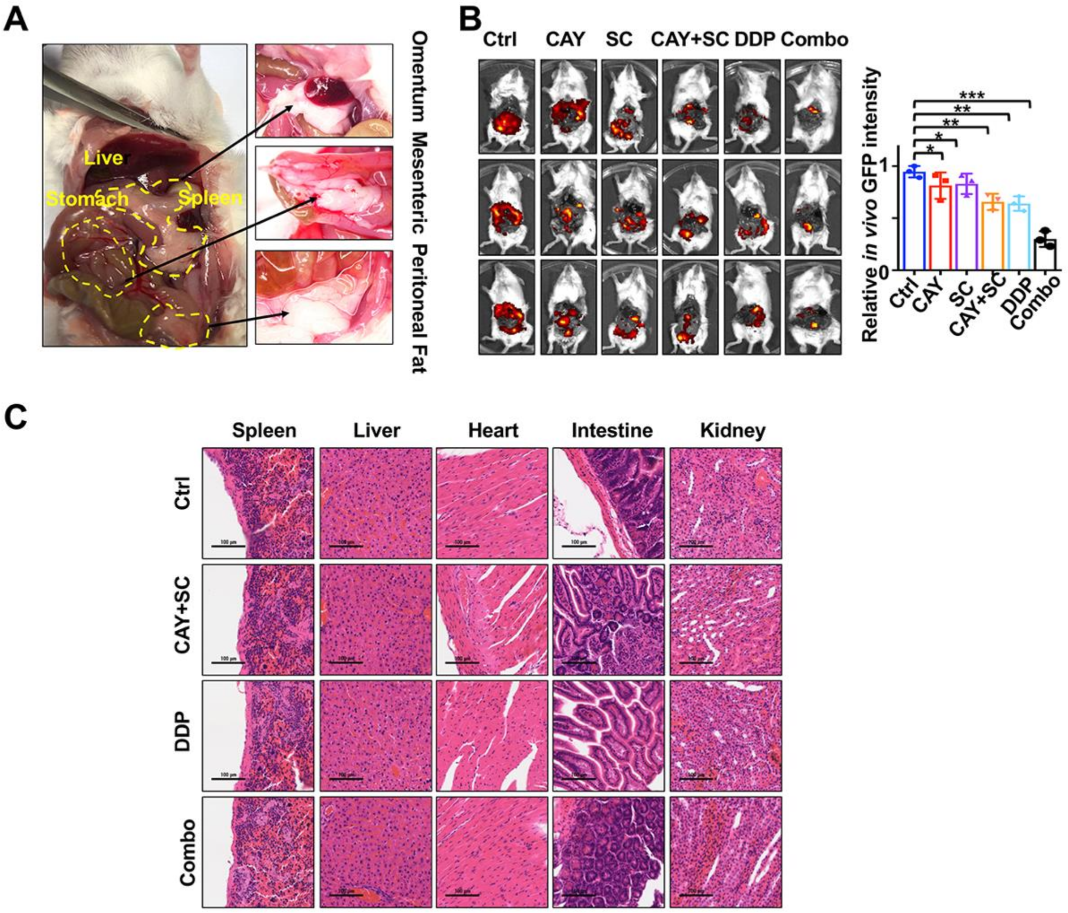

Figure S7. Combined treatment of cisplatin with inhibitors of SCD1 and FADS2 suppresses OvCa metastasis via reducing M2 aggregation
Related to Figure 7

(A) Anatomic dissection showing peritoneal fat in mice abdomen.
(B) GFP fluorescence IVIS images were obtained after the mice were sacrificed.
(C) H&E staining showed histopathology of the kidney, intestines, heart, liver, and spleen of each group of mice. Scale bar, 100 µm.

Materials Table

REAGENT or RESOURCE	SOURCE	IDENTIFIER
Antibodies		
SCD1	Cell Signaling Technology	Cat#2794S; RRID: AB_2183099
FADS2	Abcam	Cat#ab170665; RRID: AB_2756875
FADS2	ThermoFisher	Cat#PA5-87765; RRID: AB_2804393
xCT	Novus	Cat# NB300-318; RRID: AB_10000581
GPX4	Abcam	Cat#125066; RRID: AB_10973901
E-cadherin	Cell Signaling Technology	Cat# 3195; RRID: AB_2291471
Vimentin	Cell Signaling Technology	Cat#5741; RRID: AB_10695459
Ki67	Santa Cruz Biotechnology	Cat#sc-23900; RRID: AB_627859
TFR1	Abcam	Cat#ab84036; RRID: AB_10673794
GAPDH	Proteintech	Cat#10494-1-AP; RRID: AB_2263076
β-actin	ThermoFisher	Catalog # PA1-183; RRID: AB_2539914
PAX8	Santa Cruz Biotechnology	Cat# sc-81353; RRID: AB_1127048
DDK-tag	OriGene	Cat# TA150030; RRID: AB_2622253
Myc-tag	Proteintech	Cat# 16286-1-AP; RRID: AB_11182162
800CW Goat anti-Rabbit IgG	LI-COR	Cat#925-32211; RRID: AB_2651127
680RD Goat anti-Mouse IgG	LI-COR	Cat#926-68070; RRID: AB_2651128
Anti-Rabbit (Alexa Fluor® 488)	Abcam	Cat#ab150077 RRID: AB_2630356
Anti-Mouse (Alexa Fluor® 647)	Abcam	Cat#ab150115, RRID: AB_2687948
KLF4	Santa Cruz Biotechnology	Cat# sc-20691, RRID: AB_669567
Bmi1	Santa Cruz Biotechnology	Cat# sc-13519, RRID: AB_626755
ZEB1	Santa Cruz Biotechnology	Cat# sc-25388, RRID: AB_2217979
SNAIL	Cell Signaling Technology	Cat# 3879, RRID: AB_2255011
SLUG	Santa Cruz Biotechnology	Cat# sc-166476, RRID: AB_2191897
Biological Samples		
Paraffin ovarian cancer sections	Queen Mary Hospital	N/A
Fresh ascites or omentum surgically resected	Queen Mary Hospital	N/A
Chemicals, Peptides, and Recombinant Proteins		
Erastin	APExBIO	Cat#B1524
Z-VAD-FMK	Selleckchem	Catalog No. S7023
BODIPY C11	Thermo Fisher	Cat#D3861
MitoSOX	Thermo Fisher	Catalog number: M36008
MitoTracker	Thermo Fisher	Catalog number: M7514
Cisplatin (DDP)	Sigma-Aldrich	Cat#P4394

Ferrostatin-1 (Fer-1)	Sigma-Aldrich	Cat#SML0583
sc26196	Sigma-Aldrich	Cat#PZ0176
CAY10566	Cayman Chemical	Cat#944808-88-2
Collagenase type II	Thermo Fisher	Cat#17101015
DNAse	Sigma-Aldrich	Cat#D5025
Growth factor-reduced Matrigel	Corning	Cat#CB-4023C
Penicillin-Streptomycin	Thermo Fisher	Cat#15140163
GlutaMAX™ Supplement	Thermo Fisher	Cat#35050061
HEPES	Thermo Fisher	Cat#15630080
R-spondin 1	PeproTech	Cat#120-38
Noggin	PeproTech	Cat#120-10C0
EGF	PeproTech	Cat#100-15
FGF-10	PeproTech	Cat#100-26
FGF-2	PeproTech	Cat#100-18B
B27	Thermo Fisher	Cat#17504044
Nicotinamide	Sigma-Aldrich	Cat#N0636
N-acetycysteine	Sigma-Aldrich	Cat#A9165
prostaglandin E2	R&D system	Cat#2296
SB202190	Sigma-Aldrich	Cat#S7076
A83-01	Sigma-Aldrich	Cat#SML07880
Y-27632 dihydrochloride	AbMole	Cat#M1817
Propidium iodide	Sigma-Aldrich	Cat#P4170
Protease inhibitor cocktail	Sigma-Aldrich	Cat#11836170001
Phenylmethylsulfonyl fluoride	Sigma-Aldrich	Cat# 10837091001
Bpil/Bbs1	Thermo Fisher	Cat#FD1014
Nuclease-free water	Thermo Fisher	Cat#AM9932
Plasmid-Safe ATP-dependent DNase	Epicentre	Cat#E3101K
TRIzol reagent	Invitrogen	Cat#15596026
Puromycine	Sigma-Aldrich	Cat#58-58-2
LB agar	USB	Cat# 22700025
LB Broth	USB	Cat# 12780029
Tween-20	Affymetrix	Cat# 9005-64-5
Critical Commercial Assays		
Lipid Extraction Kit (Chloroform Free)	Cell Biolabs	Cat#STA-612
Lipid Quantification	Cell Biolabs	Cat#STA-613

Kit (Colorimetric)		
Membrane fluidity kit	Abcam	Cat#ab189819
Opal™ Polaris 7 Color Automation IHC Detection Kit	Akoya	Cat#NEL811001KT
Alexa Fluor 488 Annexin V/Dead Cell Apoptosis Kit	Thermo Fisher	Cat# V13241
CellEvent™ Caspase-3/7 Green Detection Reagent	Thermo Fisher	Cat#C10423
Hoechst 33342	Thermo Fisher	Cat# H3570
OxiSelect™ Intracellular ROS Assay Kit (Green Fluorescence)	Cell Biolabs	Cat#STA-342
Iron Assay Kit	ScienCell	Cat#8448
GSH/GSSG Ratio Detection Assay Kit II (Fluorometric - Green)	Abcam	Cat#ab205811
XTT cell proliferation kit	Roche	Cat#11465015001
Matrigel Invasion Chambers	Corning	Cat#354480
Migration multiwell insert plates	Corning	Cat#351157
Western blot Cell Lysis Buffer (10X)	Cell Signaling	Cat#9803
LipofectamineTM 3000 transfection reagent	Invitrogen	Cat#L3000001
QIAquick gel extraction kit	Qiagen	Cat#28704
Quickligation kit	New England Biolabs	Cat#M2200S
SuperScript™ VILO™ cDNA Synthesis Kit	Thermo Fisher	Cat#11754050
TaqMan™ Universal PCR Master Mix	Thermo Fisher	Cat#4304437
BCA Protein Assay Kit	Thermo Fisher	Cat#A53225
D-Luciferin, Potassium Salt	Gold Biotechnology	Cat#LUCK-1G

(Proven and Published^{TM})		
Seahorse XF Cell Mito Stress Test Kit	Agilent	Cat#103015-100
Western blot Cell Lysis Buffer (10X)	Cell Signaling	Cat #9803
Qiagen Plasmid Midi Kit	Qiangen	Cat#12143
Qiagen Plasmid Mini Kit	Qiangen	Cat# 12125
Qiagen Gel Purification Kit	Qiangen	Cat# 28704
10XTris/Glycine/ SDS Buffer	Bio-Rad	Cat#1610732
40%Acrylamid/B is Solution	Bio-Rad	Cat#1610148
DAPI	Thermo Fisher	Cat#D1306
RIPA Cell Lysis Buffer (10X)	Abcam	CAT#ab156034
SuperScript™ VILO™ cDNA Synthesis Kit	Thermo Fisher	Cat#11754050
TaqMan™ Universal PCR Master Mix	Thermo Fisher	Cat#4304437
Deposited Data		
TCGA-OV	This paper	TCGA
GSE168720	This paper	Gene Expression Omnibus
Experimental Models: Cell Lines		
HOSE17-1	provided by Professor G. S. W. Tsao, School of Biomedical Sciences, The University of Hong Kong.	No STR reference for comparison immortalized cell line
HOSE11-12	provided by Professor G. S. W. Tsao, School of Biomedical Sciences, The University of Hong Kong.	No STR reference for comparison immortalized cell line
HOSE96-9-18	provided by Professor G. S. W. Tsao, School of Biomedical Sciences, The University of Hong Kong.	No STR reference for comparison immortalized cell line
A2780s	provided by Prof. Benjamin Tsang, University of Ottawa, Canada	RRID: CVCL_0134
A2780cp	provided by Prof. Benjamin Tsang, University of Ottawa, Canada	RRID: CVCL_0135
PEO1	Sigma-Aldrich	10032308 RRID: CVCL_2686
PEO4	Sigma-Aldrich	10032309 RRID: CVCL_2690
SK-OV-3	ATCC	HTB-77 RRID: CVCL_0532
OVCA433	provided by Professor G. S. W. Tsao, School of Biomedical	RRID: CVCL_0475

	Sciences, The University of Hong Kong.	
ES-2	ATCC	CRL-1978 RRID: CVCL_3509
OVCAR-3	ATCC	HTB-161 RRID: CVCL_0465
OVKATE	JCRB Cell Bank	JCRB1044 RRID: CVCL_3110
OVSAHO	JCRB Cell Bank	JCRB1046 RRID: CVCL_3114
COV318	Sigma-Aldrich	07071903 RRID: CVCL_2419
T HESCs	ATCC	CRL-4003 RRID: CVCL_C464
ID8	provided by Dr.Katherine Roby, University of Kansas Medical Center	No STR reference for comparison Murine OvCa cell line
THP-1	ATCC	TIB-202 RRID: CVCL_0006
Experimental Models: Organisms/Strains		
C.B-17/Icr-scid (SCID)	Charles River Lab, USA	https://www.lau.hku.hk/en/Animals/Animals/Animal-Strains
Oligonucleotides		
SCD1 sgRNA1	5′-CATAAGGACGATATCCGAAG-3′;	
SCD1 sgRNA2	5′-GCCGAGCTTTGTAAGAGCGG-3′	
SCD1 sgRNA3	5′-ATGTCGTCTTCCAAGTAGAG-3′	
FADS2 sgRNA1	5′-CTTACACAAGATCGCCCCGC-3′	
FADS2 sgRNA2	5′-CTTGTCCACAAATTCGTCAT-3′	
FADS2 sgRNA3	5′-AGAACTTGCCCACGAATTCC-3	
Recombinant DNA		
Myc-DDK-tagged SCD plasmid	OriGene	RC209108
Myc-DDK tagged FADS2	OriGene	RC223780
SCD1 shRNA Lentiviral Particle	Santa Cruz	sc-36465-V
FADS2 shRNA Lentiviral Particle	Santa Cruz	sc-96449-V
vector PX459 (pSpCas9(BB)-2A-Puro V2.0)	Addgene	Cat#62988
vector PX458(SpCas9-2A-GFP) Plasmid	Addgene	Cat#48138

Software and Algorithms		
GraphPad Prism version 8	GraphPad Software	https://www.graphpad.com/scientific-software/prism/
R studio	R Core Team	www.r-project.org
Biorender	Biorender	https://biorender.com/
real-time PCR	Thermo Fisher	ViiA 7 Real-Time PCR System
CompuSyn	ComboSyn	https://www.combosyn.com/
LI-COR Odyssey CLx Imaging System	LI-COR Biosciences	CLX-0983
Confocal Microscope	Carl Zeiss	LSM 800
FlowJo_10.6.1_CL	FlowJo	https://www.flowjo.com/
ImageJ	National Institute of Health	https://imagej.nih.gov/ij/
DESeq2	CL FlowJo (Love et al., 2014)	https://www.flowjo.com http://bioconductor.org/packages/release/bioc/html/DESeq2.html
ZOE™ Fluorescent Imager	Bio-Rad,Hercules, CA, USA	https://www.bio-rad.com/en-hk/product/zoe-fluorescent-cell-imager?ID=N74CIZE8Z
IVIS® Spectrum In Vivo Imaging System	PerkinElmer	https://www.perkinelmer.com
